# Supplementary material for: Spatial dynamics of CD39+CD8+ exhausted T cell reveal tertiary lymphoid structures-mediated response to PD-1 blockade in esophageal cancer
Source: Nat Commun. 2024 Oct 19;15:9033. doi: 10.1038/s41467-024-53262-w (PMC11490492; doi:10.1038/s41467-024-53262-w)
Supplement: Supplementary file 1 — Supplementary Information [file 41467_2024_53262_MOESM1_ESM.pdf]

Supplementary Information

Spatial dynamics of CD39^{+}CD8^{+} exhausted T cell reveal tertiary lymphoid structures-mediated response to PD-1 blockade in esophageal cancer

Kenro Tanoue[1], Hirofumi Ohmura[1, 2], Koki Uehara[1], Mamoru Ito[1], Kyoko Yamaguchi[1], Kenji Tsuchihashi[1], Yudai Shinohara[3], Peng Lu[4], Shingo Tamura[5], Hozumi Shimokawa[6], Taichi Isobe[1, 2], Hiroshi Ariyama[7], Yoshihiro Shibata[8], Risa Tanaka[9], Hitoshi Kusaba[6], Taito Esaki[10], Kenji Mitsugi[11], Daisuke Kiyozawa[12], Takeshi Iwasaki[12], Hidetaka Yamamoto[12,13], Yoshinao Oda[12], Koichi Akashi[1], Eishi Baba [2,*]

1. Department of Medicine and Biosystemic Science, Graduate School of Medical Sciences, Kyushu University, Fukuoka, Japan
2. Department of Oncology and Social Medicine, Graduate School of Medical Sciences, Kyushu University, Fukuoka, Japan
3. Department of Hematology/Oncology, Japan Community Healthcare Organization Kyushu Hospital, Fukuoka, Japan
4. Department of Imaging Science Program, McKelvey School of Engineering, Washington University in St. Louis, Missouri, USA
5. Department of Medical Oncology, NHO National Hospital Organization Kyushu Medical Center, Fukuoka, Japan
6. Department of Medical Oncology, Hamanomachi Hospital, Fukuoka, Japan
7. Department of Medical Oncology, Kitakyushu Municipal Medical Center, Fukuoka, Japan
8. Department of Medical Oncology, Fukuoka Wajiro Hospital, Fukuoka, Japan

9. Department of Medical Oncology, St Mary's Hospital, Kurume, Japan

10. Department of Gastrointestinal and Medical Oncology, National Kyushu Cancer Center, Fukuoka, Japan

11. Department of Medical Oncology, Sasebo Kyosai Hospital, Nagasaki, Japan

12. Department of Anatomic Pathology, Graduate School of Medical Sciences, Kyushu University, Fukuoka, Japan

13. Department of Pathology, Dentistry and Pharmaceutical Sciences, Graduate School of Medicine, Okayama University, Okayama, Japan

*Corresponding author

Supplementary Figures

Supplementary Fig. 1. Characteristics of CD8$^+$ T cell phenotypes from acquired region of interests (ROIs) and their association with clinical outcomes Supplementary Fig.2: Spatial mapping of unsupervised CD8$^+$ T cell clusters.

Supplementary Fig. 3: The identification of CD8$^+$ T cell phenotypes within tumor and secondary lymphoid organ (SLO) from mass flow cytometry (MC) dataset.

Supplementary Fig.4: Identification of PD-1$^+$ CD8$^+$ T cells in blood.

Supplementary Fig. 5: CD39$^+$Ki67$^+$PD-1$^+$CD8$^+$ T (CD39$^+$Ki67$^+$ Tex) cell population in the blood is phenotypically exhausted and relatively abundant in cancer patients compared to healthy donors.

Supplementary Fig.6: Phenotypic and functional differences between proliferative CD39$^+$ Tpex and CD39$^+$ dTex populations in the blood.

Supplementary Fig.7: Identification of CD39$^+$ Tpex cells within SLOs

Supplementary Fig.8: Visualized IMC images for all available samples.

Supplementary Fig. 9: Representative expression of SLO and tumor markers.

Supplementary Fig. 10: Cell identification workflow for IMC.

Supplementary Fig. 11: Comparison of the denoised IMC images versus immunofluorescence-stained images.

Supplementary Fig. 12: Gating strategy for cell type identification using IMC.

Supplementary Tables

Supplementary Table 1: Patient characteristics.

Supplementary Table 2: List of tumor panel for imaging mass cytometry.

Supplementary Table 3: List of SLO panel for imaging mass cytometry.

Supplementary Table 4: List of panel for mass flow cytometry.

Supplementary Table 5: List of key reagents

Supplementary Fig. 1. Characteristics of CD8^{+} T cell phenotypes from acquired region of interests (ROIs) and their association with clinical outcomes.

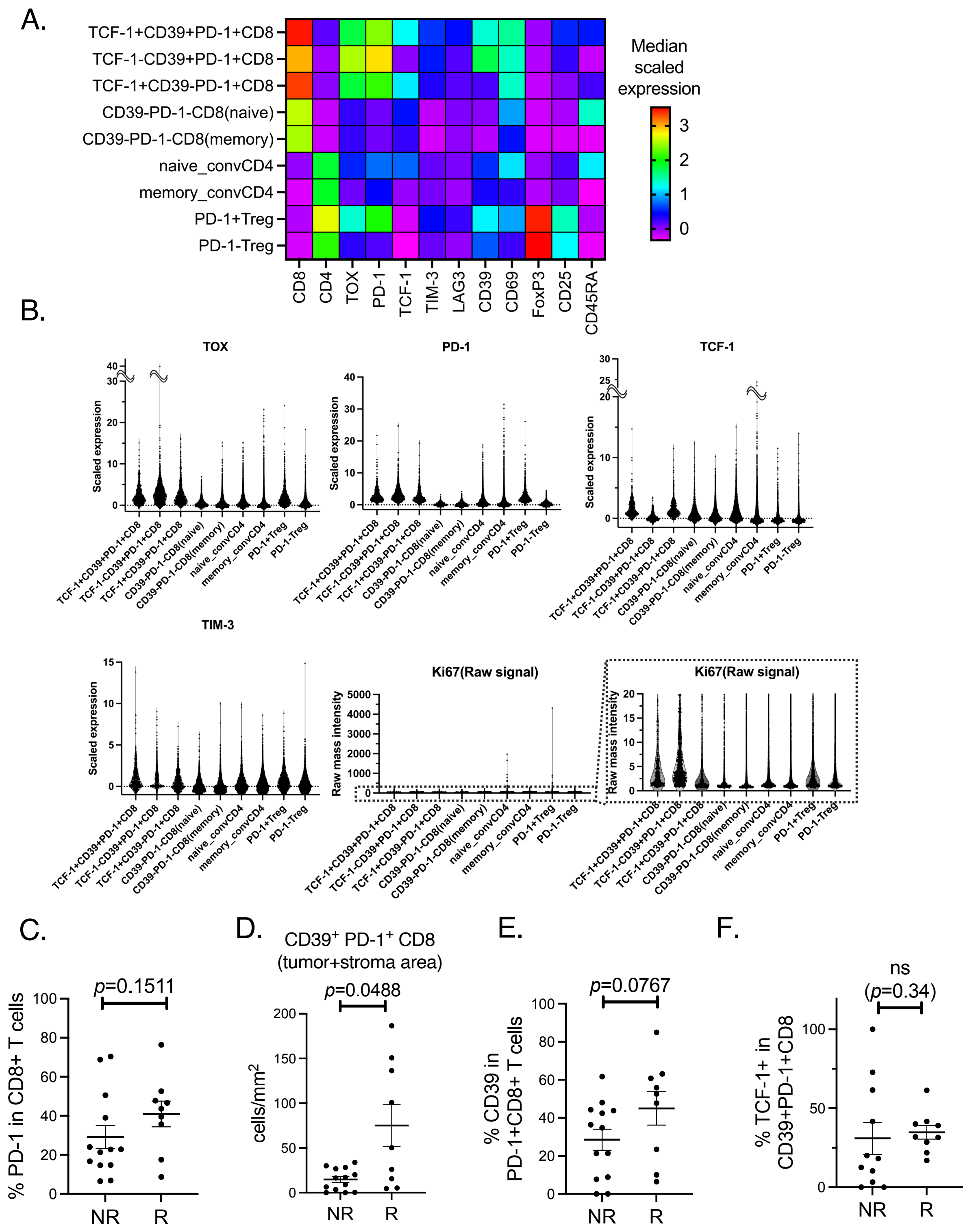

(A) Heatmap colored by median scaled expression in each indicated T cell type from all ROIs at pre-treatment. Each cell type was identified according to B: Imaging mass cytometry (IMC) image processing analysis (Fig. S11B). (B) Violin plots of scaled marker expression (and raw signal of Ki67) in each indicated T cell phenotype. (C) Fraction (%) of cells expressing PD-1 among $CD8^+$ T cells for responders (Rs) (n=9) and non-responders (NRs) (n=13) (Rs vs. NRs: P=0.1511). (D) Density of $CD39^+PD\text{-}1^+CD8^+$ T cells among Rs (n=9) and NRs (n=13) (Rs vs. NRs: P=0.0488). (E) Fraction (%) of cells expressing CD39 among $PD\text{-}1^+CD8^+$ T cells for Rs (n=9) and NRs (n=13) (Rs vs. NRs: P=0.0767). (F) Fraction (%) of cells expressing TCF-1 among $PD\text{-}1^+CD8^+$ T cells for Rs (n=9) and NRs (n=11) (Rs vs. NRs: P=0.34). Two cases were excluded due to cold tumors lacking $CD39^+PD\text{-}1^+CD8^+$ T cells. (C, D, E, F) Five NE cases are excluded from these analyses. Two-sided Mann-Whitney *U*-test. n refers to independent tumor-ROIs. Error bars indicate mean ± SEM. Source data are provided as a Source Data file.

Supplementary Fig. 2. Spatial mapping of unsupervised CD8^{+} T cell clusters.

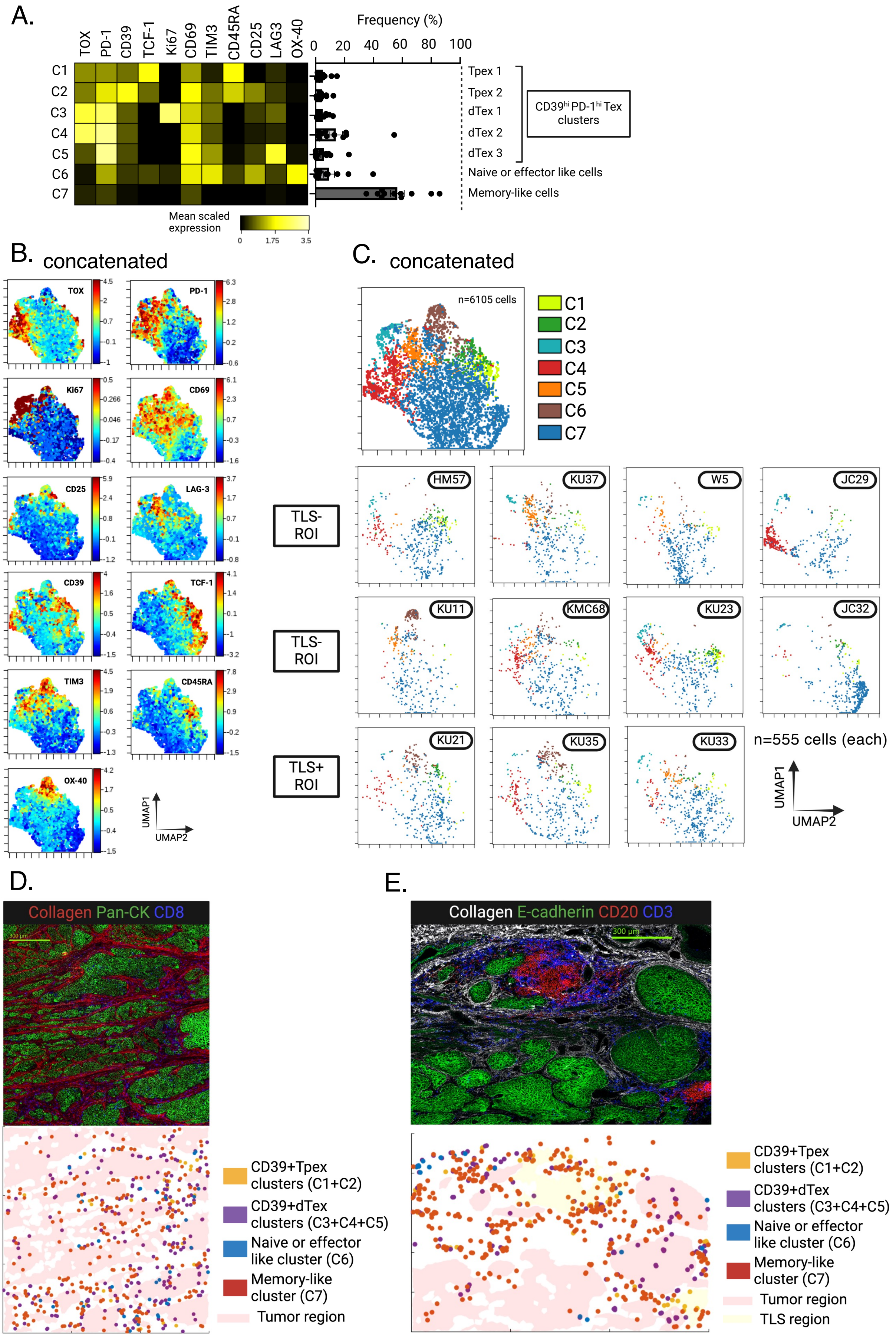

(A) Heatmap colored by mean scaled expression for each CD8$^+$ T cell cluster (left). Frequency of each cluster from 11 individual ROIs before ICB treatment (right) (B) UMAP plots of concatenated CD8$^+$ T cells (n= 6105 cells) from 11 ROIs (left). (C) Unsupervised clusters visualized in the UMAP plots for concatenated CD8$^+$ T cells (n = 6105 cells) and individual CD8$^+$ T cells (each 555 cells) from 11 ROIs. Clusters are color-coded as follows: C1: Yellow, C2: Green, C3: Light blue, C4: Red, C5: Orange, C6: Brown, C7: Blue. (D) The representative ROI lacking TLSs, colored according to collagen (red), Pan-cytokeratin (PanCK) (green), and CD8 (blue) (top). Among the 555 CD8$^+$ T cells, classified clusters are mapped onto the corresponding left image classified by tumor areas (parenchyma). CD39$^+$ precursor exhausted T (Tpex) clusters (C1+C2) are shown in orange, CD39$^+$ differentiated exhausted T (dTex) clusters (C3+C4+C5) in purple, Naive or effector-like clusters (C6) in blue, Memory-like clusters (C7) in red, and Tumor regions in light pink (bottom). (E) The representative tumor-ROI including TLSs, colored according to collagen (white), E-cadherin (green), CD20 (red), and CD3 (blue) (top). Among 555 CD8$^+$ T cells, classified clusters are mapped onto the corresponding left image classified by the tumor, stroma and TLS areas. CD39$^+$ Tpex clusters (C1+C2) are shown in orange, CD39$^+$ dTex clusters (C3+C4+C5) in purple, Naive or effector-like clusters (C6) in blue, Memory-like clusters (C7) in red, Tumor regions in light pink, and TLS regions in light yellow (bottom). (D, E) Similar distribution patterns are confirmed among independent 11 ROIs, each containing over 500 CD8$^+$ T cells. (A) Error bars indicate mean ± SEM. (A, C) were created in BioRender. Kenro, T. (2024) BioRender.com/o01b035. Source data are provided as a Source Data file.

Supplementary Fig. 3. The identification of CD8^{+} T cell phenotypes within tumor and secondary lymphoid organ (SLO) from mass flow cytometry (MC) dataset.

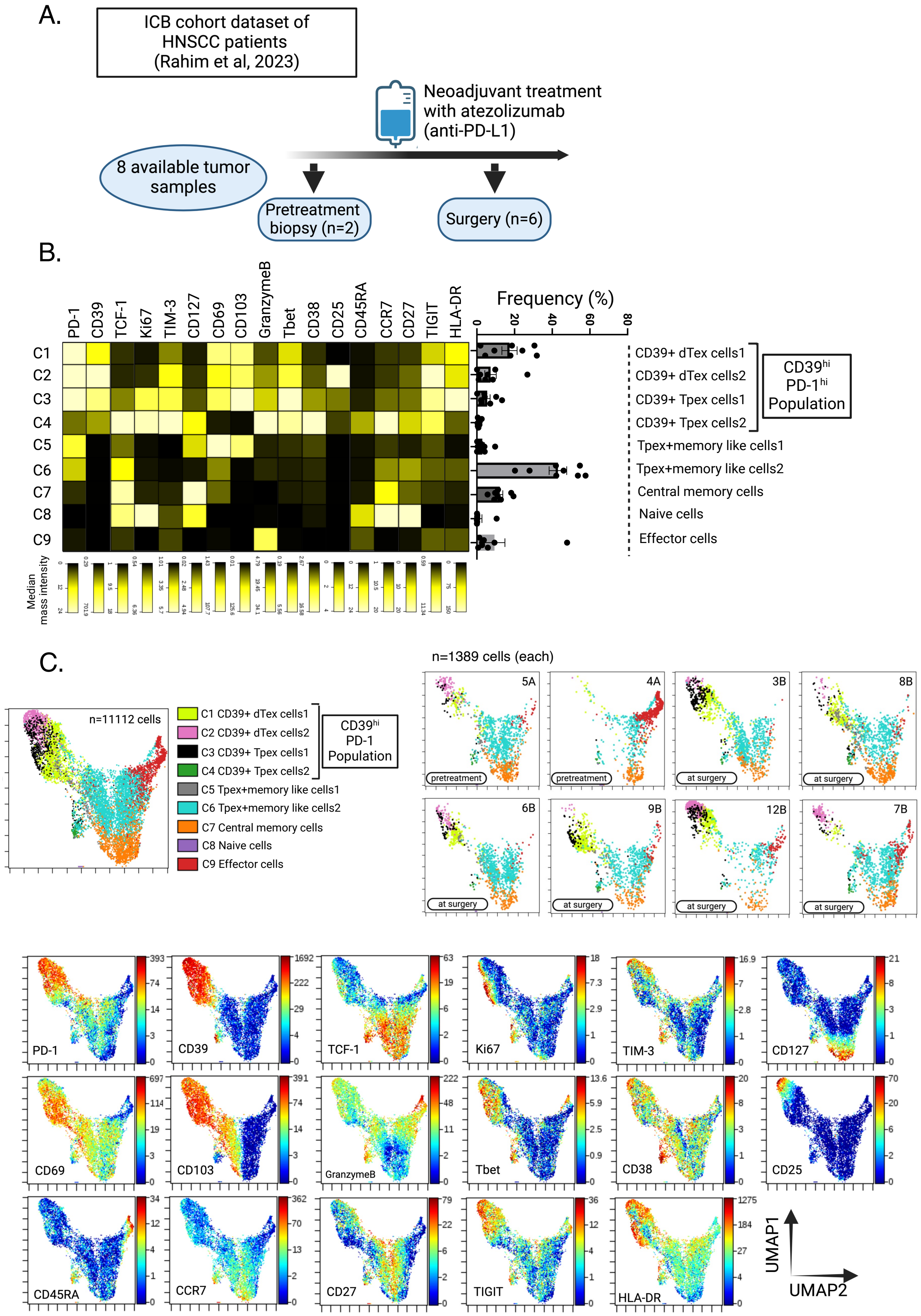

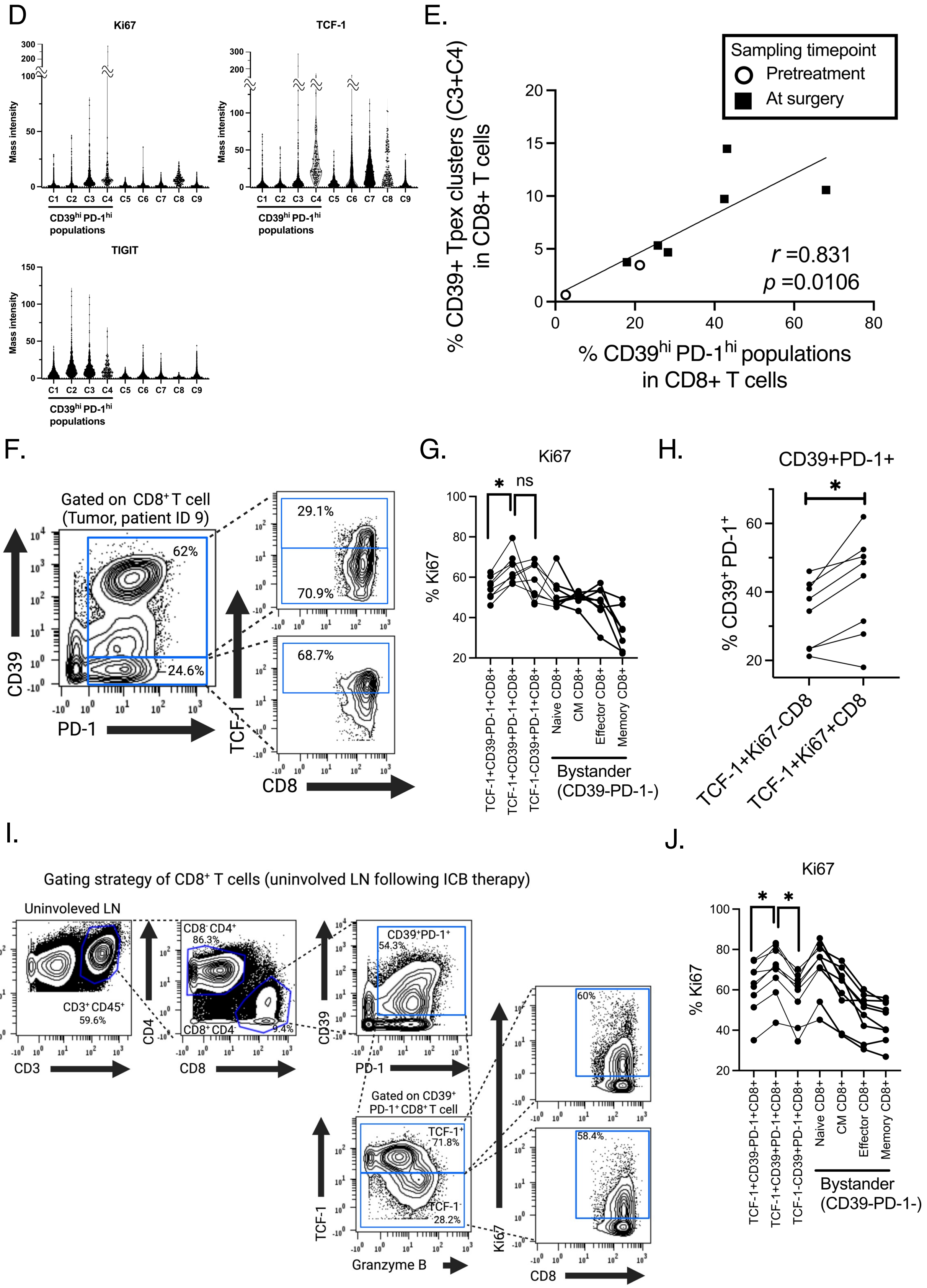
D
Ki67
TCF-1
TIGIT
Mass intensity
C1 C2 C3 C4 C5 C6 C7 C8 C9
CD39hi PD-1hi populations
E.
Sampling timepoint
Pretreatment
At surgery
% CD39+ Tpex clusters (C3+C4) in CD8+ T cells
% CD39hi PD-1hi populations in CD8+ T cells
r =0.831
p =0.0106
F.
Gated on CD8+ T cell (Tumor, patient ID 9)
62%
24.6%
29.1%
70.9%
68.7%
CD39
PD-1
TCF-1
CD8
G.
Ki67
% Ki67
ns
TCF-1+CD39-PD-1+CD8+
TCF-1+CD39+PD-1+CD8+
TCF-1-CD39+PD-1+CD8+
Naive CD8+
CM CD8+
Effector CD8+
Memory CD8+
Bystander (CD39-PD-1-)
H.
CD39+PD-1+
% CD39+ PD-1+
TCF-1+Ki67-CD8
TCF-1+Ki67+CD8
I.
Gating strategy of CD8+ T cells (uninvolved LN following ICB therapy)
Uninvoleved LN
CD3+ CD45+
59.6%
CD8- CD4+
86.3%
CD8+ CD4-
9.4%
CD39+PD-1+
54.3%
Gated on CD39+ PD-1+ CD8+ T cell
TCF-1+
71.8%
TCF-1-
28.2%
60%
58.4%
CD45
CD3
CD4
CD8
CD39
PD-1
TCF-1
Granzyme B
Ki67
J.
Ki67
% Ki67
TCF-1+CD39-PD-1+CD8+
TCF-1+CD39+PD-1+CD8+
TCF-1-CD39+PD-1+CD8+
Naive CD8+
CM CD8+
Effector CD8+
Memory CD8+
Bystander (CD39-PD-1-)

(A) Schematic illustration of 8 individual tumor samples in the immune checkpoint blockade (ICB) cohort dataset of HNSCC patients. (B) Heatmap colored by median mass intensity of marker expression in each of 9 indicated $CD8^+$ T cell clusters (left). Frequency (%) of each $CD8^+$ T cell cluster (right). (C) Unsupervised clusters visualized in the UMAP plots for concatenated $CD8^+$ T cells (11112 cells) and individual $CD8^+$ T cells (each 1389 cells) from 8 individual tumor samples. Clusters are color-coded as follows: C1 $CD39^+$ dTex cells1: yellow, C2 $CD39^+$ dTex cells2: pink, C3 $CD39^+$ Tpex cells1: black, C4 $CD39^+$ Tpex cells2: green, C5 Tpex+memory like cells1: gray, C6 Tpex+memory like cells2: light blue, C7 Central memory cells: orange, C8 Naive cells: purple, C9 Effector cells: red (top). UMAP plots colored by the expression of multiple markers in concatenated $CD8^+$ T cells (11112 cells) from 8 individual tumor samples (bottom). (D) Violin plots showing the mass intensity of marker expression in each cluster of concatenated $CD8^+$ T cells (11112 cells). (E) Frequency (%) of $CD39^+$ Tpex clusters (C3+C4) in $CD8^+$ T cells relative to that of $CD39^{hi}PD\text{-}1^{hi}$ clusters (C1+C2+C3+C4) in $CD8^+$ T cells (n=8 independent tumor samples); Pearson's correlations using a two-sided test (r=0.831, P=0.0106). (F) The manual gating of each $CD8^+$ T cell subset from a representative tumor sample. (G) Fraction (%) of each type of T cell expressing Ki67 from the eight independent tumor samples of ICB cohort dataset. The gating strategy for Naive, Central Memory (CM), Effector, and Effector Memory (Memory) T cells is based on the expression of CD45RA or CCR7; repeated measures analysis of variance (ANOVA), followed by post-hoc tests with Bonferroni correction ($TCF\text{-}1^+CD39^-PD\text{-}1^+CD8^+$ T cells vs. $TCF\text{-}1^+CD39^+PD\text{-}1^+CD8^+$ T cells: P=0.0005, $TCF\text{-}1^+CD39^+PD\text{-}1^+CD8^+$ T cells vs. $TCF\text{-}1^-CD39^+PD\text{-}1^+CD8^+$ T cells: P=0.54). (H) Fraction (%) of $TCF\text{-}1^+Ki67^+CD8^+$ and $TCF\text{-}1^+Ki67^-CD8^+$ T cells co-expressing CD39 and PD-1 from the eight independent tumor samples of ICB cohort dataset; two-sided Wilcoxon matched-pairs signed test ($TCF\text{-}1^+Ki67^+CD8^+$ vs. $TCF\text{-}1^+Ki67^-CD8^+$ T cells: P=0.016). (I) The manual gating of each $CD8^+$ T cell subset from an uninvolved lymph node (LN) of an ICB-treated patient from ICB cohort dataset. Only one sample was obtained in this cohort. (J)

Fraction (%) of each type of T cell expressing Ki67 from the nine independent LN samples of standard of care cohort dataset; repeated measures ANOVA followed by post-hoc tests with Bonferroni correction (TCF-1^{+}CD39^{-}PD-1^{+}CD8^{+} T cells vs. TCF-1^{+}CD39^{+}PD-1^{+}CD8^{+} T cells; P=0.0003, TCF-1^{+}CD39^{+}PD-1^{+}CD8^{+} T cells vs. TCF-1^{-}CD39^{+}PD-1^{+}CD8^{+} T cells; P=0.0011). *P<0.05. (B) Error bars indicate mean ± SEM. (A, B, C) were created in BioRender. Kenro, T. (2024) BioRender.com/o01b035. Source data are provided as a Source Data file.

Supplementary Fig. 4. Identification of PD-1^{+}CD8^{+} T cells in blood.

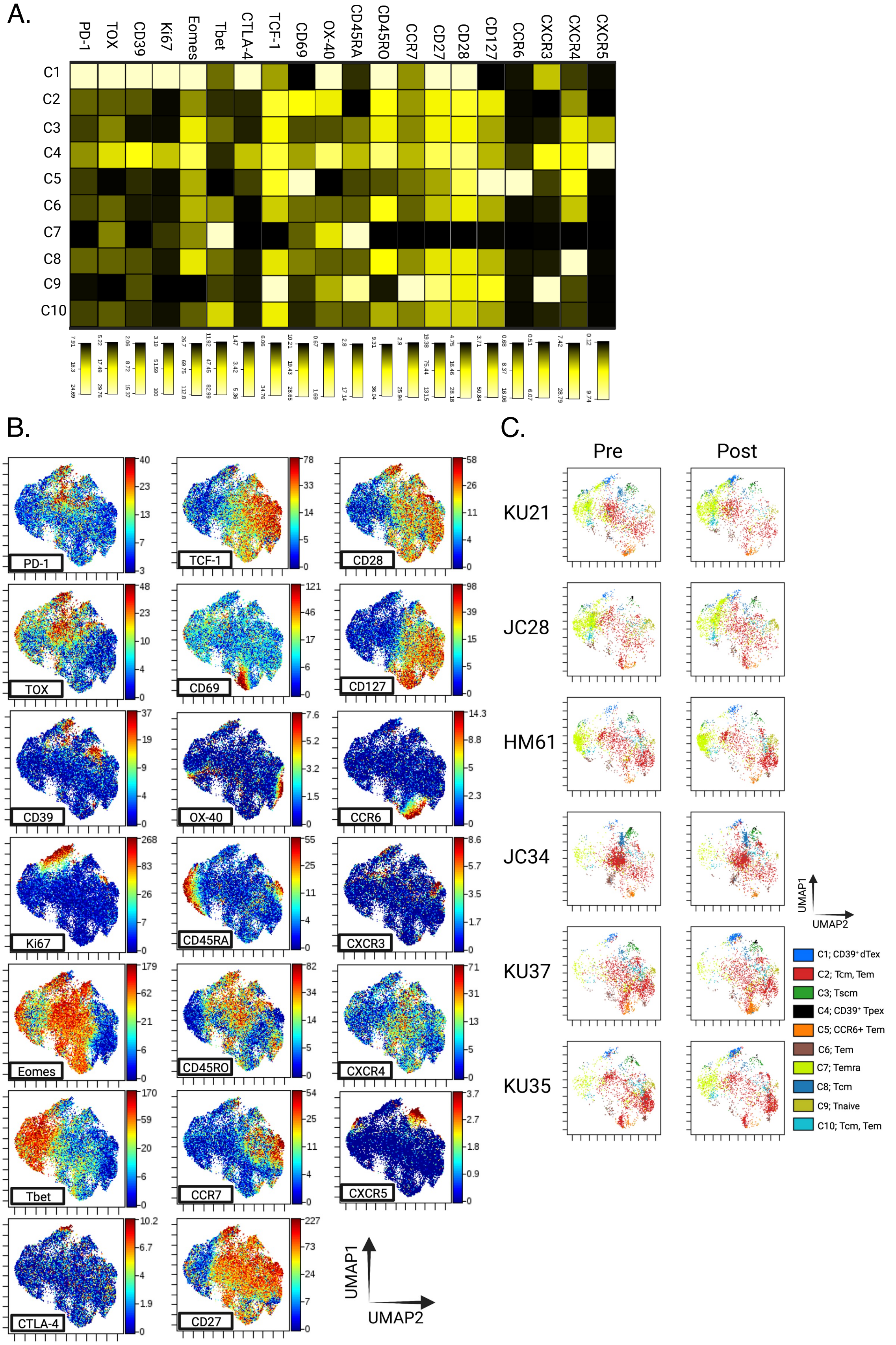

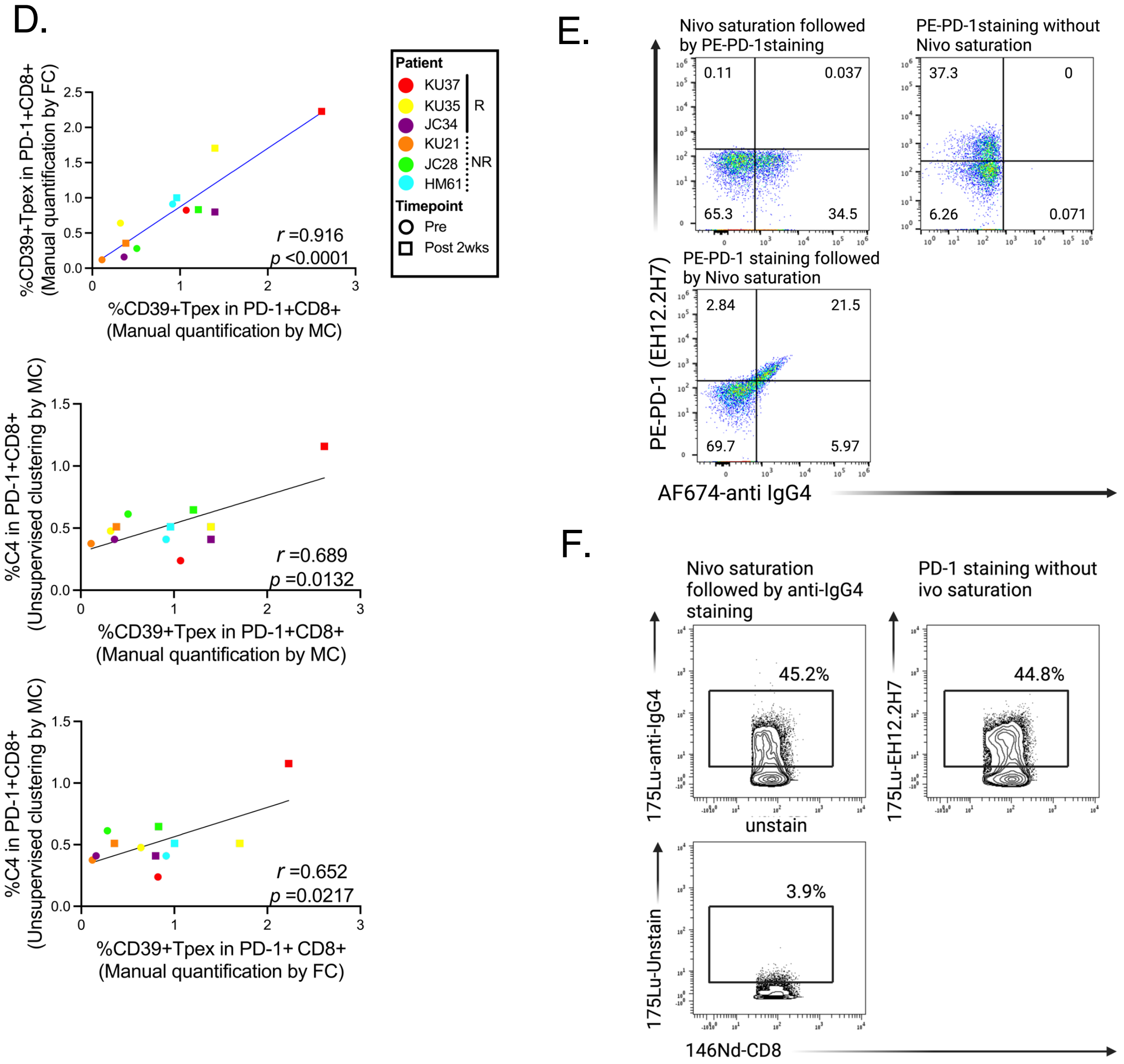

(A) Heatmap colored by mean mass intensity of marker expression in each of 10 indicated clusters from 12 independent peripheral blood mononuclear cell (PBMC) samples. (B) UMAP plots colored by individual markers for concatenated PD-1$^+$CD8$^+$ T cells from 12 PBMC samples. (C) Individual PD-1$^+$CD8$^+$ T cell clusters (2,936 cells per sample) visualized in UMAP plots from 6 patients at pre- and post-treatment. (D) Comparison of proliferative CD39$^+$ Tpex cells in blood at pre- and post-treatment, quantified by manual gating by flow cytometry (FC) (% TCF-1$^+$CD39$^+$Ki67$^+$), vs. manual gating by MC (% TCF-1$^+$CD39$^+$Ki67$^+$), vs. unsupervised clustering by MC (% C4) (n=12 PBMC samples); Pearson's correlations using a two-sided test (manual gating by FC vs. manual gating by MC: r= 0.916, P<0.0001, manual gating by FC vs. unsupervised clustering by MC: r=0.652, P=0.0217,

manual gating by MC vs. unsupervised clustering by MC: r=0.689, P=0.0132). (E) Healthy donor PBMCs were incubated with nivolumab for 30 min, followed by a subsequent 30-min incubation with anti–PD-1 clone EH12.2H7 PE and then another 30-min incubation with anti-human IgG4 AF647 (left-upper). Healthy donor PBMCs were incubated for 30 min with anti–PD-1 clone EH12.2H7 PE without nivolumab saturation and then for another 30 min with anti-human IgG4 AF647 (right-upper). Healthy donor PBMCs were incubated with anti–PD-1 clone EH12.2H7 PE for 30 min followed by a subsequent 10-min incubation with nivolumab and then another 30-min incubation with anti-human IgG4 AF647 (lower left). (F) Healthy donor PBMCs were incubated with nivolumab for 30 min, followed by a 30-min incubation with anti-human IgG4 175 Lu (upper left). Healthy donor PBMCs were incubated for 30 min with anti–PD-1 clone EH12.2H7 175Lu (upper right). Healthy donor PBMCs without staining (lower left). Source data are provided as a Source Data file.

Supplementary Fig. 5. CD39$^+$Ki67$^+$PD-1$^+$CD8$^+$ T (CD39$^+$Ki67$^+$ Tex) cell population in the blood is phenotypically exhausted and relatively abundant in cancer patients compared to healthy donors.

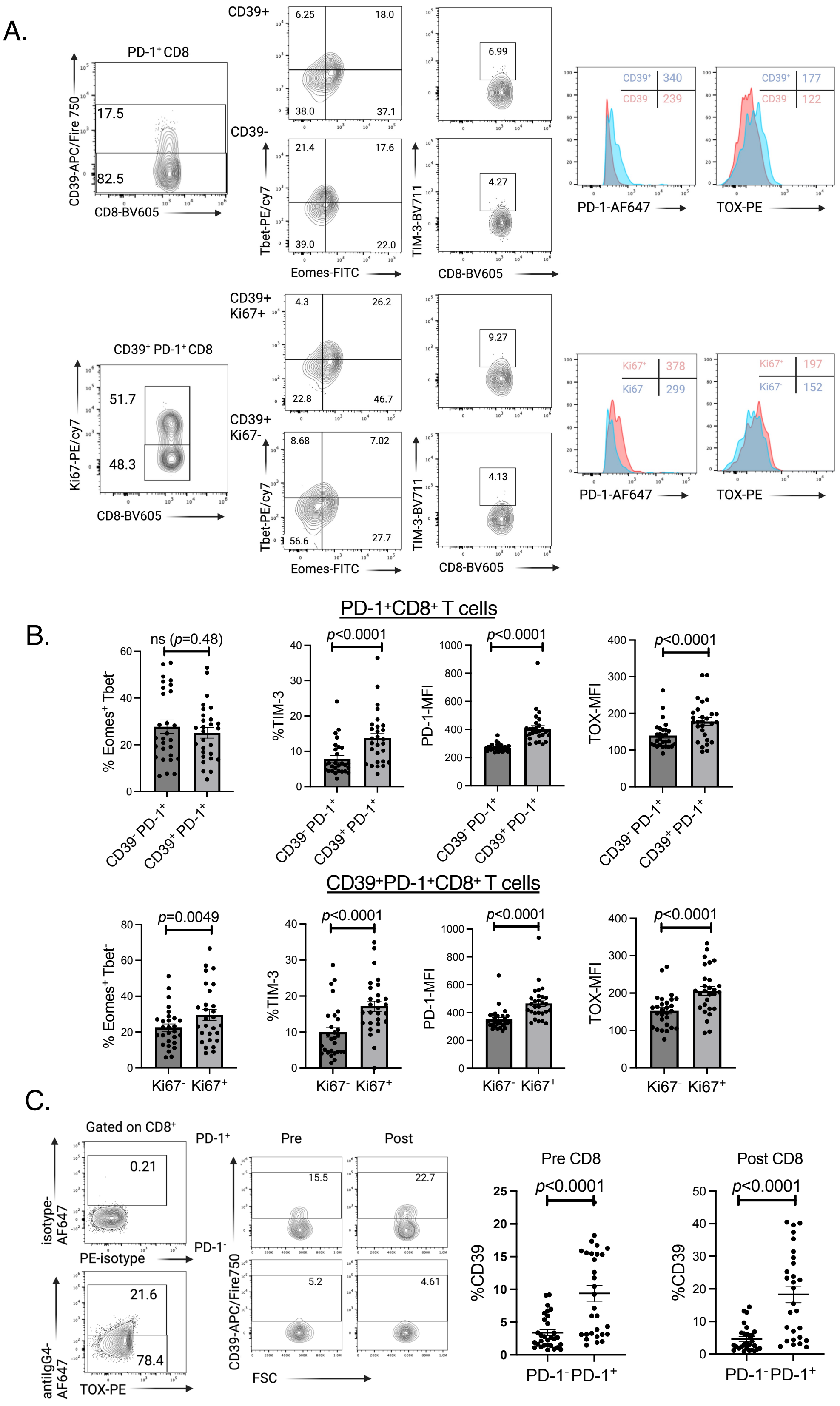

D.

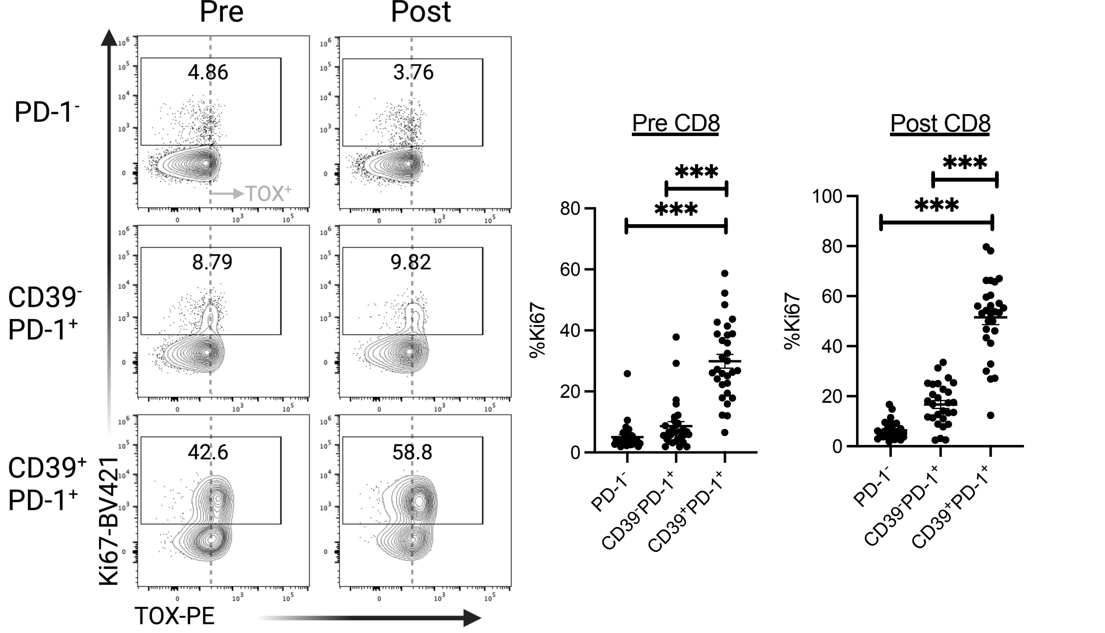

E.

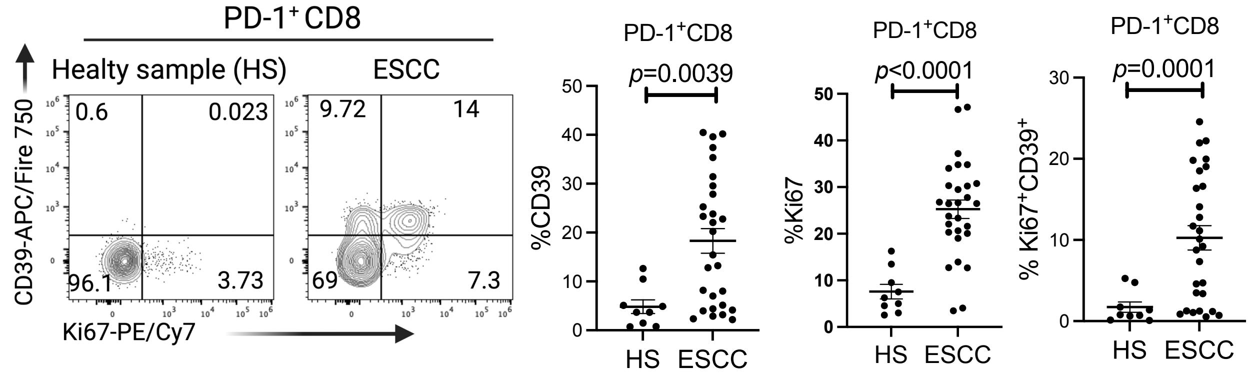

(A) Representative plots of CD39$^+$PD-1$^+$ and CD39$^-$PD-1$^+$CD8$^+$ T cells (top). Representative plots of CD39$^+$Ki67$^+$PD-1$^+$ and CD39$^+$Ki67$^-$PD-1$^+$CD8$^+$ T cells (bottom). (B) Dysfunctional marker expression in CD39$^-$PD-1$^+$ and CD39$^+$PD-1$^+$CD8$^+$ T cells; two-sided Wilcoxon matched-pairs test (n=30, CD39$^-$PD-1$^+$ vs. CD39$^+$PD-1$^+$CD8$^+$ T cells) (top). Dysfunctional marker expression in CD39$^+$Ki67$^-$PD-1$^+$ and CD39$^+$Ki67$^+$PD-1$^+$CD8$^+$ T cells; two-sided Wilcoxon matched-pairs test (n=30, CD39$^+$Ki67$^-$PD-1$^+$ vs. CD39$^+$Ki67$^+$PD-1$^+$CD8$^+$ T cells) (bottom). (C) Fraction (%) of cells expressing CD39 among PD-1$^+$CD8$^+$ T cells and PD-1$^-$CD8$^+$ T cells pre- and post-treatment; two-sided Wilcoxon matched-pairs test (pre: n=30, post: n=28). (D) Fraction (%) of cells expressing Ki67 among PD-1$^-$, CD39$^-$PD-1$^+$, and CD39$^+$PD-1$^+$ CD8$^+$ T cells from 30 patients at pre-ICB and 28

patients at post-ICB; repeated measures ANOVA followed by post-hoc tests with Bonferroni correction (PD-1^{-} vs. CD39^{-}PD-1^{+} vs. CD39^{+}PD-1^{+} CD8^{+} T cells). (E) Fraction (%) of CD39^{+}, Ki67^{+}, and CD39^{+}Ki67^{+} population among PD-1^{+}CD8^{+} T cells in nine healthy samples (HS) and 28 ESCC patients; two-sided Mann-Whitney *U*-test (HS vs. ESCC patients). ***P<0.0001. (B, C, D. E) Error bars indicate mean ± SEM. (B, C) n refers to independent PBMC samples (A, C, D, E) The staining patterns of the FCS plots were compared across all samples with similar results. Source data are provided as a Source Data file.

Supplementary Fig. 6. Phenotypic and functional differences between proliferative CD39$^+$ Tpex and CD39$^+$ dTex populations in the blood.

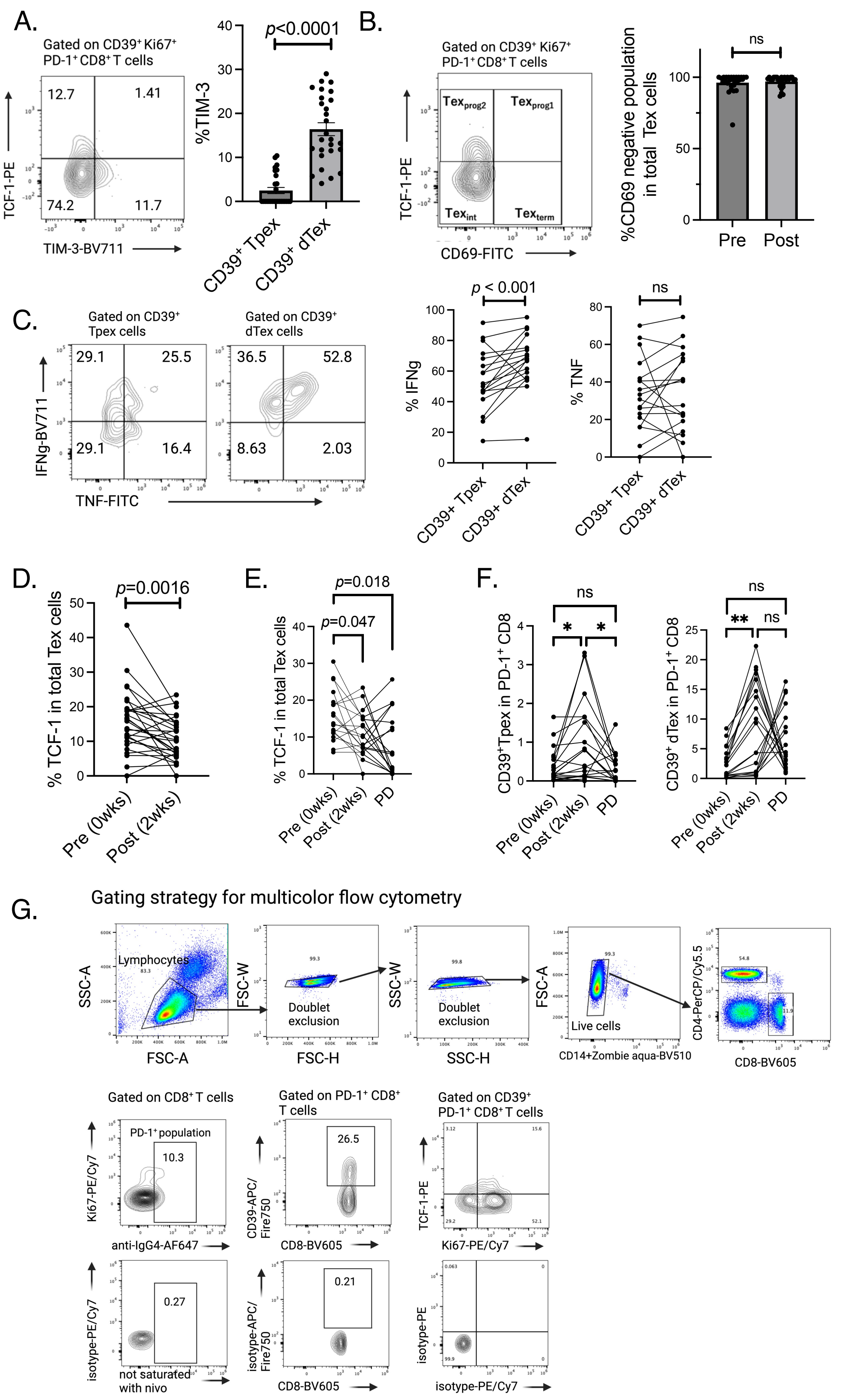

(A) Fraction (%) of cells expressing TIM-3 among $CD39^+$ Tpex and $CD39^+$ dTex cells at pre-ICB (n=30); Two-sided Wilcoxon matched-pairs test. (B) Fraction (%) of CD69-negative population among $CD39^+Ki67^+PD\text{-}1^+CD8^+$ T ($CD39^+Ki67^+$ Tex) cells pre- and post-treatment; two-sided Wilcoxon matched-pairs test (n=28, pre vs. post: P=0.72). (C) Fraction (%) of cells expressing IFN-γ and TNF among $CD39^+$ Tpex and $CD39^+$ dTex cells at pre-treatment; two-sided Wilcoxon matched-pairs test (each n=18, IFN-γ: P=0.0008, TNF: P=0.39). 18 patients were randomly selected. (D) Fraction (%) of cells expressing TCF-1 among $CD39^+$ Tpex cells pre- and post-treatment; two-sided Wilcoxon matched-pairs test (n=28, pre vs. post: P=0.0016). (E) Fraction (%) of cells expressing TCF-1 among $CD39^+$ Tpex cells pre- and post-treatment and upon PD (n=19); repeated measures ANOVA followed by post-hoc tests with Bonferroni correction (each n=19, pre vs. post: P=0.047, pre vs. PD: P=0.018). (F) Fraction (%) of $CD39^+$ Tpex and $CD39^+$ dTex cells among $PD\text{-}1^+$ $CD8^+$ T cells pre- and post-treatment and upon PD; repeated measures ANOVA followed by post-hoc tests with Bonferroni correction (each n=19, $CD39^+$ Tpex; pre vs. post: P=0.037, post vs. PD: P=0.038, pre vs. PD: P>0.99, $CD39^+$ dTex; pre vs. post: P=0.0004, post vs. PD: P>0.99, pre vs. PD: P=0.11). (G) Gating strategy for identifying proliferative $CD39^+$ Tpex cells and $CD39^+$ dTex cells. The upper panel represents the lineage gating, while the lower panel shows the gating criteria for these cells using isotype controls. *P<0.05, **P<0.01. (A, B) Error bars indicate mean ± SEM. (A, B, C, D, E, F) n refers to independent PBMC samples. (A, B, C) The staining patterns of the FCS plots were compared across all available samples with similar results. Source data are provided as a Source Data file.

Supplementary Fig. 7. Identification of CD39^{+} Tpex cells within SLOs

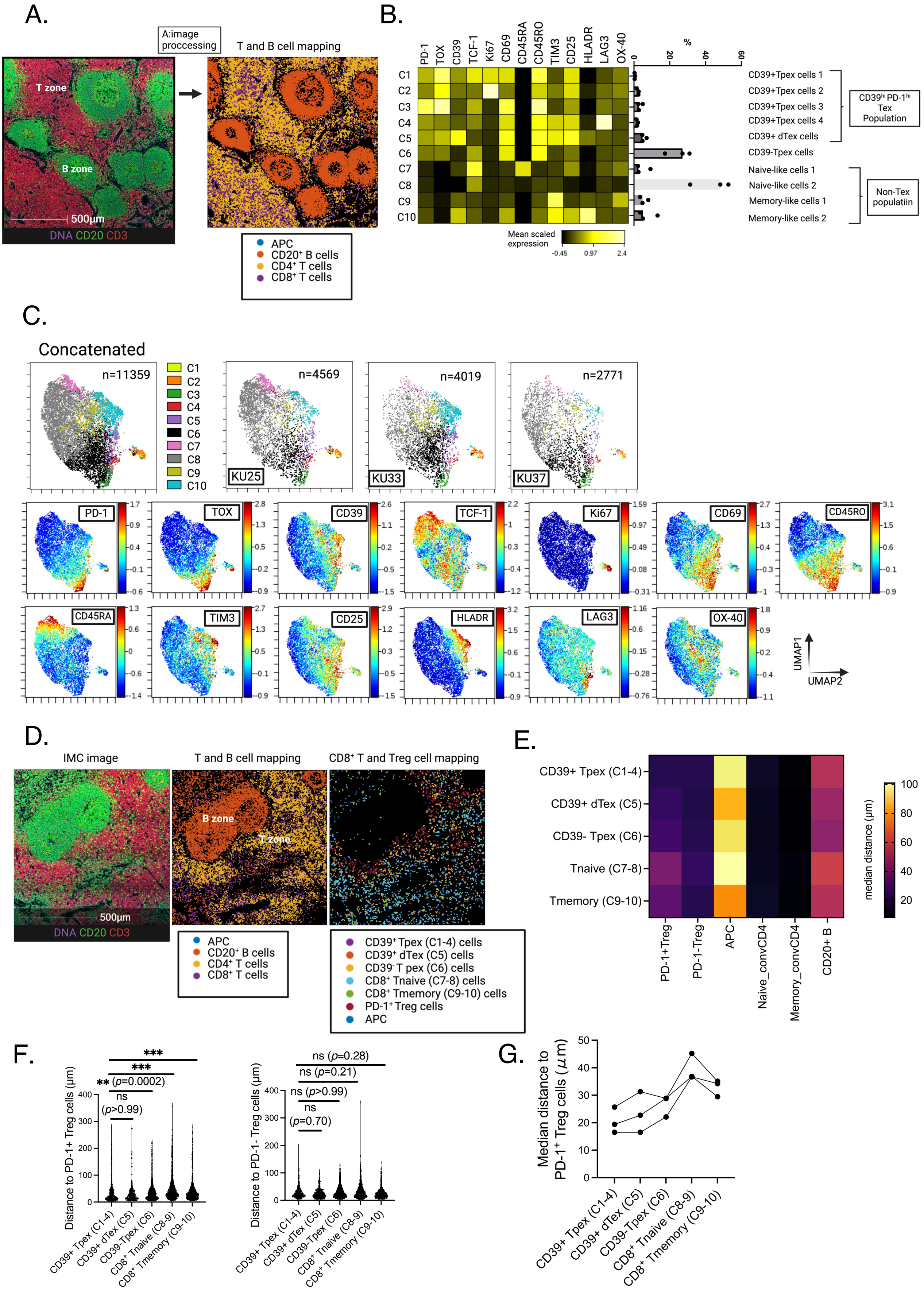

(A) The representative SLO-ROI, colored according to DNA (purple), CD20 (green), and CD3 (red) (Patient ID KU33) (left). Spatial mapping of the major immune cell types from the corresponding left image. The colors of plots are as follows: antigen-presenting cells (APC): blue, $CD20^+$ B cells: orange, $CD4^+$ T cells: yellow, $CD8^+$ T cells: purple (right). (B) Heatmap colored by mean scaled expression in each $CD8^+$ T cell cluster within the three independent SLO-ROIs at pre-treatment (left). Frequency (%) of each $CD8^+$ T cell cluster (right). (C) Unsupervised $CD8^+$ T cell clusters visualized in the UMAP plots for concatenated $CD8^+$ T cells (11359 cells) and individual $CD8^+$ T cells from three SLO samples (top). UMAP plots of concatenated $CD8^+$ T cells (11359 cells) from three SLO samples (bottom). (D) The left image shows the IMC image of the representative SLO-ROI, colored according to DNA (purple), CD20 (green), and CD3 (red) (Patient ID KU37). The middle image illustrates the spatial mapping of the major immune cell types within this SLO-ROI. The colors of plots are as follows: APC: blue, $CD20^+$ B cells: orange, $CD4^+$ T cells: yellow, $CD8^+$ T cells: purple. The right image provides a selective mapping of $CD8^+$ T cell clusters, PD-1^+ Treg cells, and APC within the corresponding SLO-ROI. The colors are as follows: $CD39^+$ Tpex (C1-4) cells: purple, $CD39^+$ dTex (C5) cells: orange, $CD39^+$ Tpex (C6) cells: red, $CD8^+$ Tnaive (C7-8) cells: light blue, $CD8^+$ Tmemory (C9-10) cells: green, PD-1^+ Treg cells: dark red, APC: blue. (E) The heatmap shows the median distance (μm) from CD8+ T cell clusters (columns) to major immune cell types (rows) across three SLO-ROIs. The median distance for each case was calculated separately, and the heatmap reflects these median distances for each individual case. (F) Distance from $CD8^+$ T cell clusters to PD-1^+ Treg (left) and PD-1^- Treg (right) cells in the representative SLO image (Patient ID KU33). The proximity of each cell in clusters C1-4 (n=285 cells), C5 (n=284 cells), C6 (n=1247 cells), C7-8 (n=1354 cells), and C9-10 (n=849 cells) to Tregs was calculated, and a comparison of these proximities was performed; Kruskal Wallis test with post-hoc Dunn comparisons. (G) Median distance from $CD8^+$ T cell clusters to PD-1^+ Treg cells within each SLO (n=3 independent SLO samples). $^{**}P<0.001$, $^{***}P<0.0001$. (A, D) Staining patterns and single-cell masks were compared for three independent

SLO-ROIs with similar results. (B) Box plots indicate median values. (B, C) were created in BioRender. Kenro, T. (2024) BioRender.com/o01b035 and BioRender.com/g70j848. Source data are provided as a Source Data file.

Supplementary Fig. 8. Visualized IMC images for all available samples.

A.

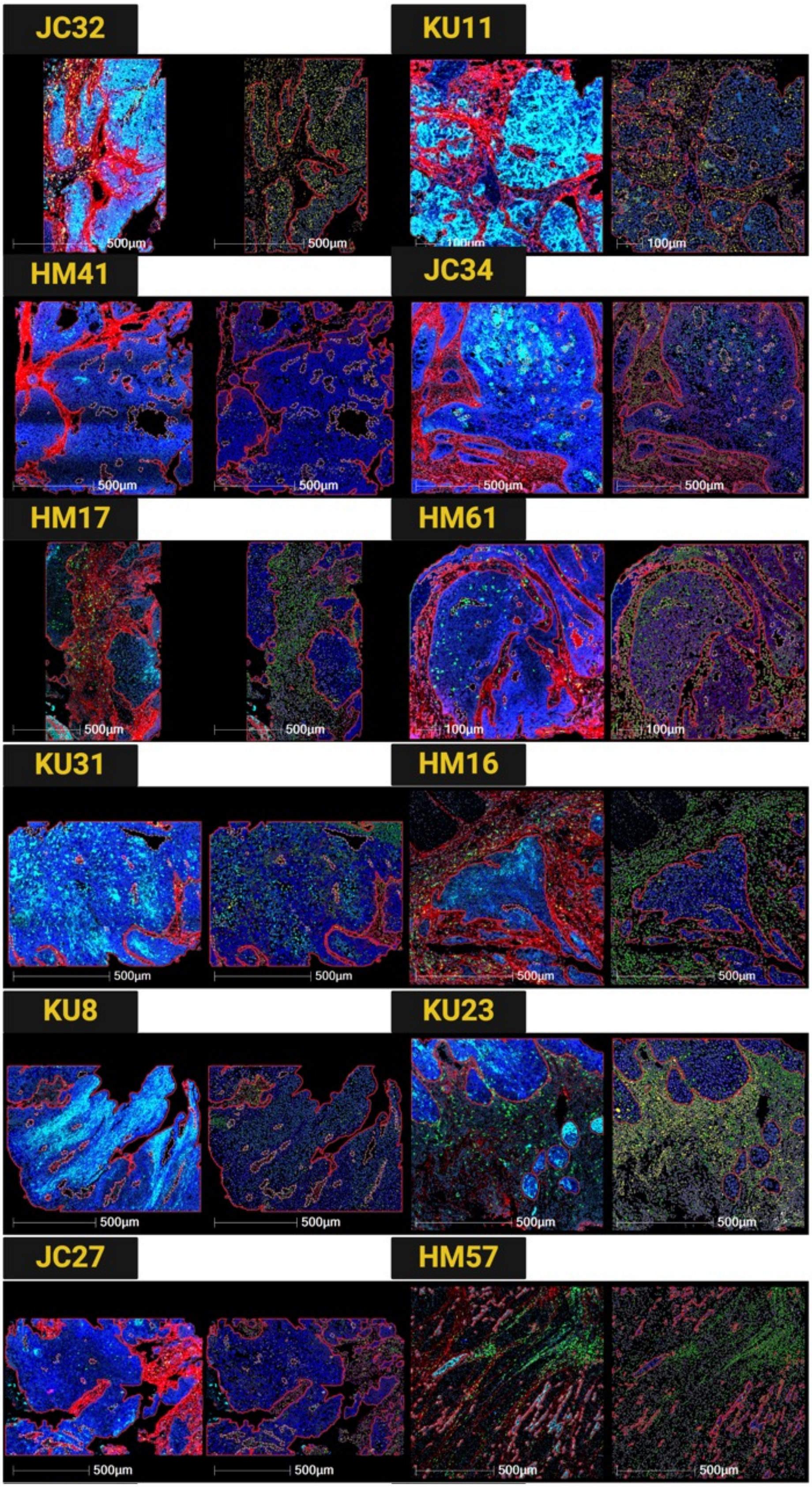

A.

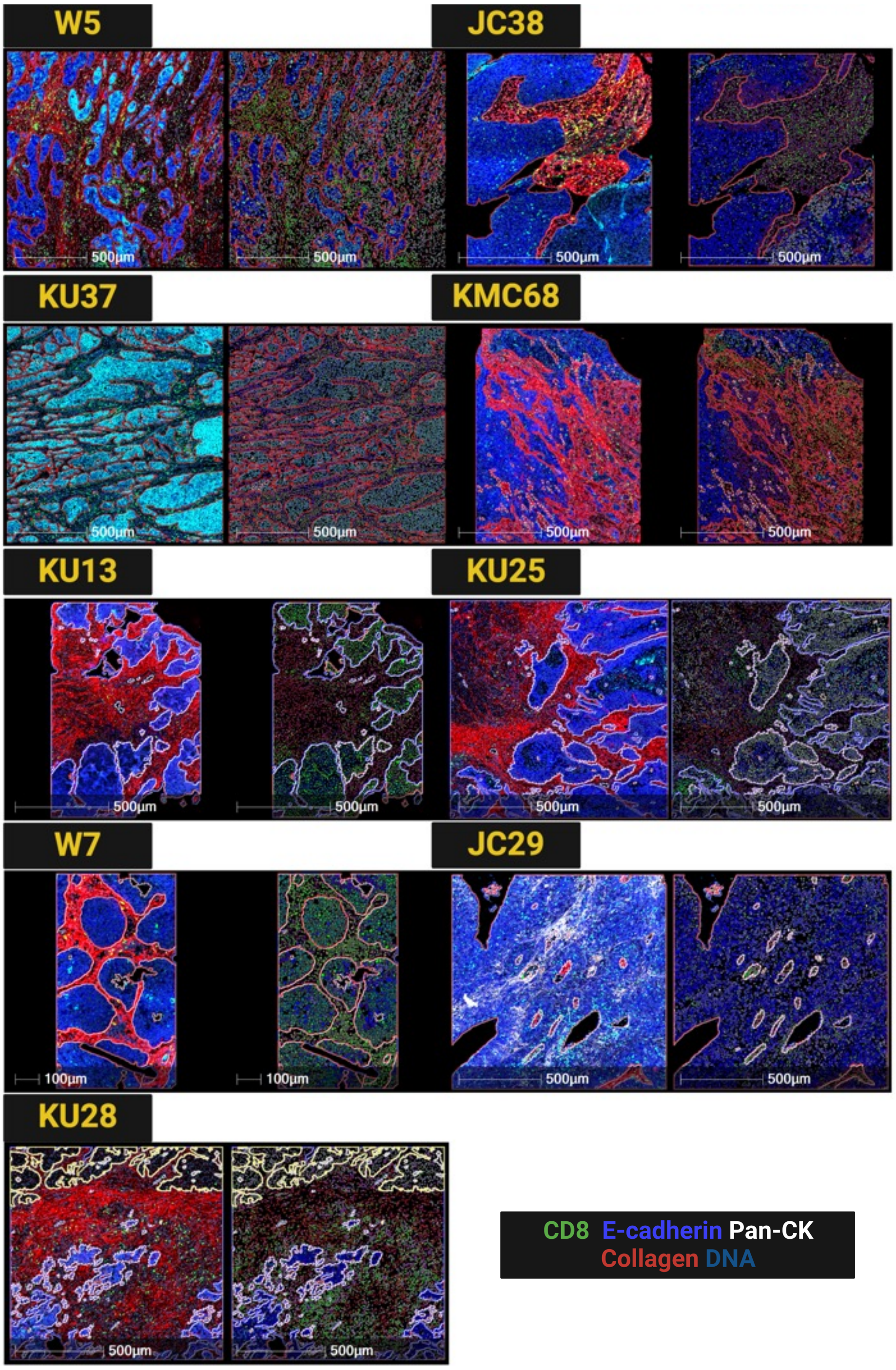
W5
JC38
KU37
KMC68
KU13
KU25
W7
JC29
KU28
500µm
100µm
CD8 E-cadherin Pan-CK
Collagen DNA

B.

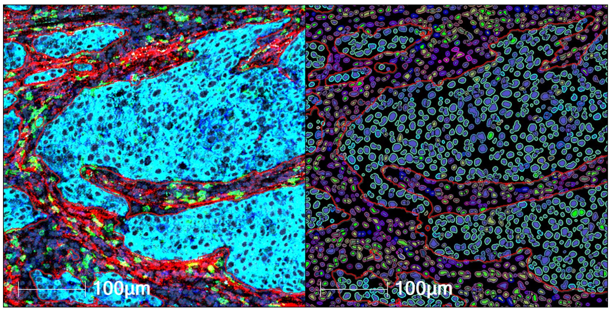
100µm
100µm

C.

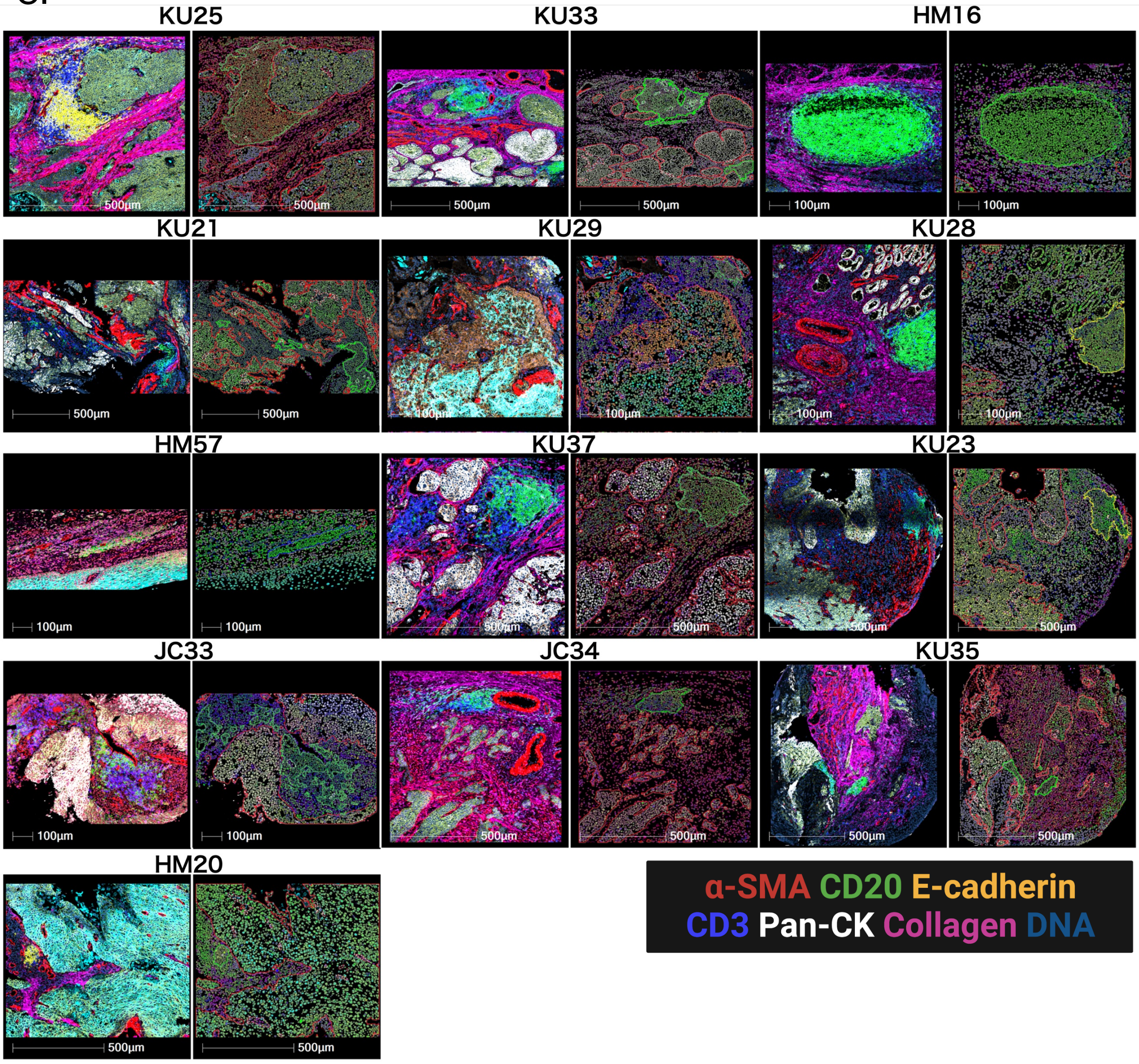
KU25
KU33
HM16
KU21
KU29
KU28
HM57
KU37
KU23
JC33
JC34
KU35
HM20
500µm
100µm
α-SMA CD20 E-cadherin
CD3 Pan-CK Collagen DNA

D.

TLS (HM20)

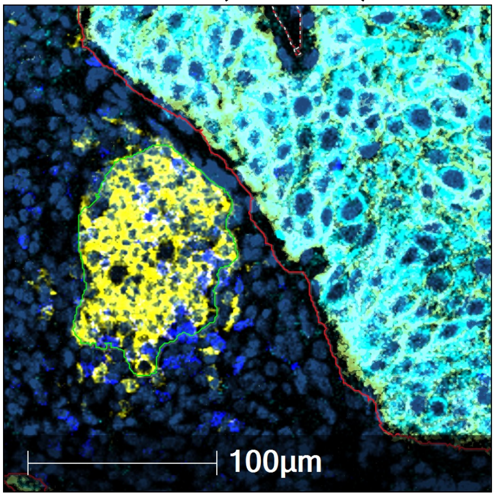
100µm

(A) Left panels show IMC images of all tumor samples without TLSs, colored for CD8 (green), E-cadherin (blue), Pan-CK (white), collagen (red), and DNA (dark blue). Right images show graphical representations corresponding to left images according to B: IMC image processing analysis (Fig. S10C). Each cell in these graphics is represented as a single cell annotated using the phenotyping strategy (Fig. S11B) according to the Halo module (Indica Labs) Highplex FL. Also, lines drawn within these graphics classify the parenchyma and stroma according to the subsequent module Halo Tissue classifier. (B) Enlarged IMC image and graphical representation corresponding to left images according to B: IMC image processing analysis (KU37). (C) Left images show IMC images of all tumor samples harboring TLSs, colored according to α-SMA (red), CD20 (green), E-cadherin (yellow), CD3 (blue), Pan-CK (white), collagen (pink), and DNA (dark blue). Each cell in these graphics is represented as a single cell annotated according to the phenotyping strategy (Fig. S11B) with the Highplex FL module. Also, lines drawn within these graphics identify the TLSs according to the Tissue classifier module. (D) Enlarged TLS image excluded from detailed TLS analysis due to small size.

Supplementary Fig. 9. Representative expression of SLO and tumor markers

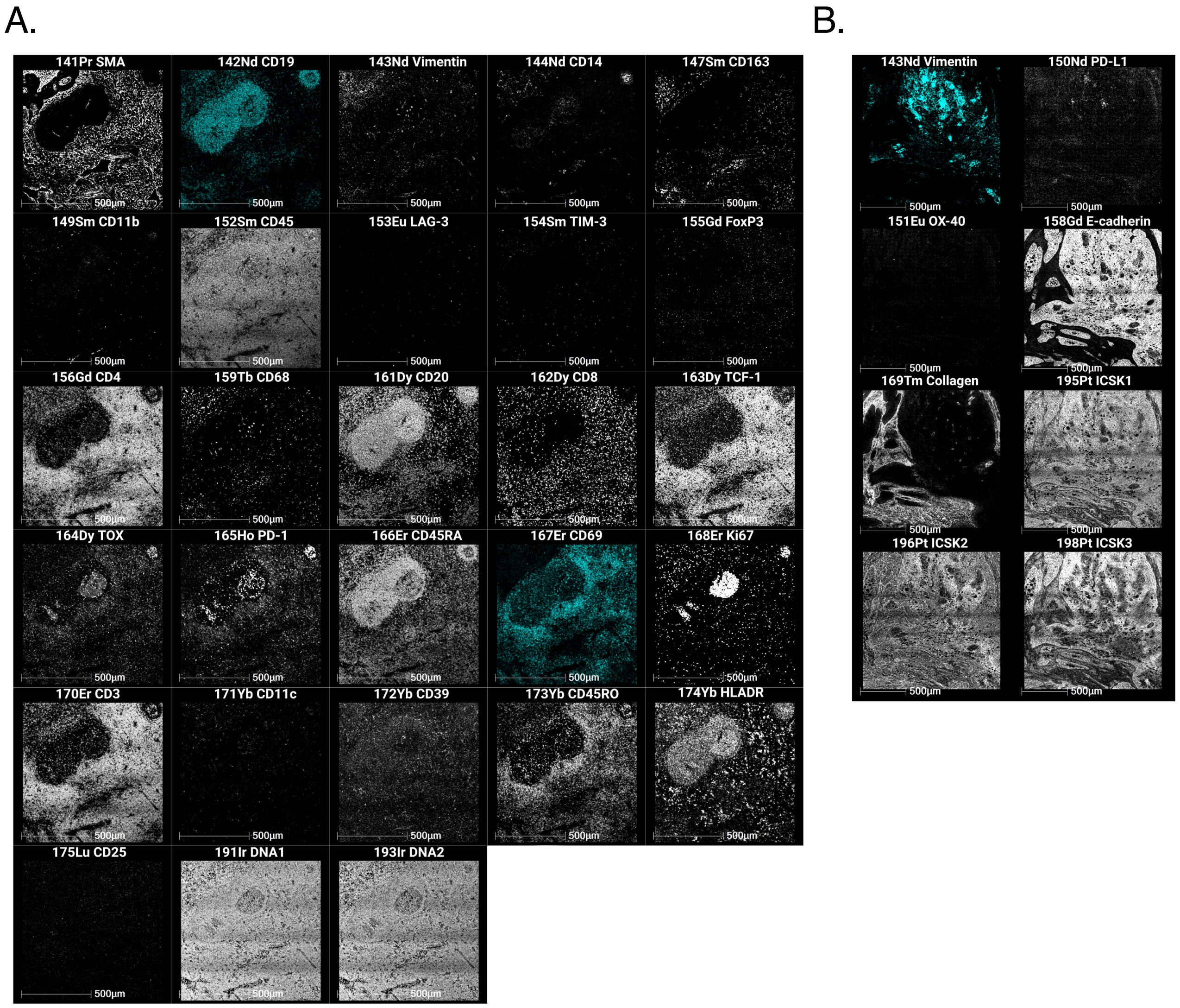

(A) Individual marker positive for SLOs (KU37). (B) Individual marker positive for tumors (JC34).

Supplementary Fig. 10. Cell identification workflow for IMC

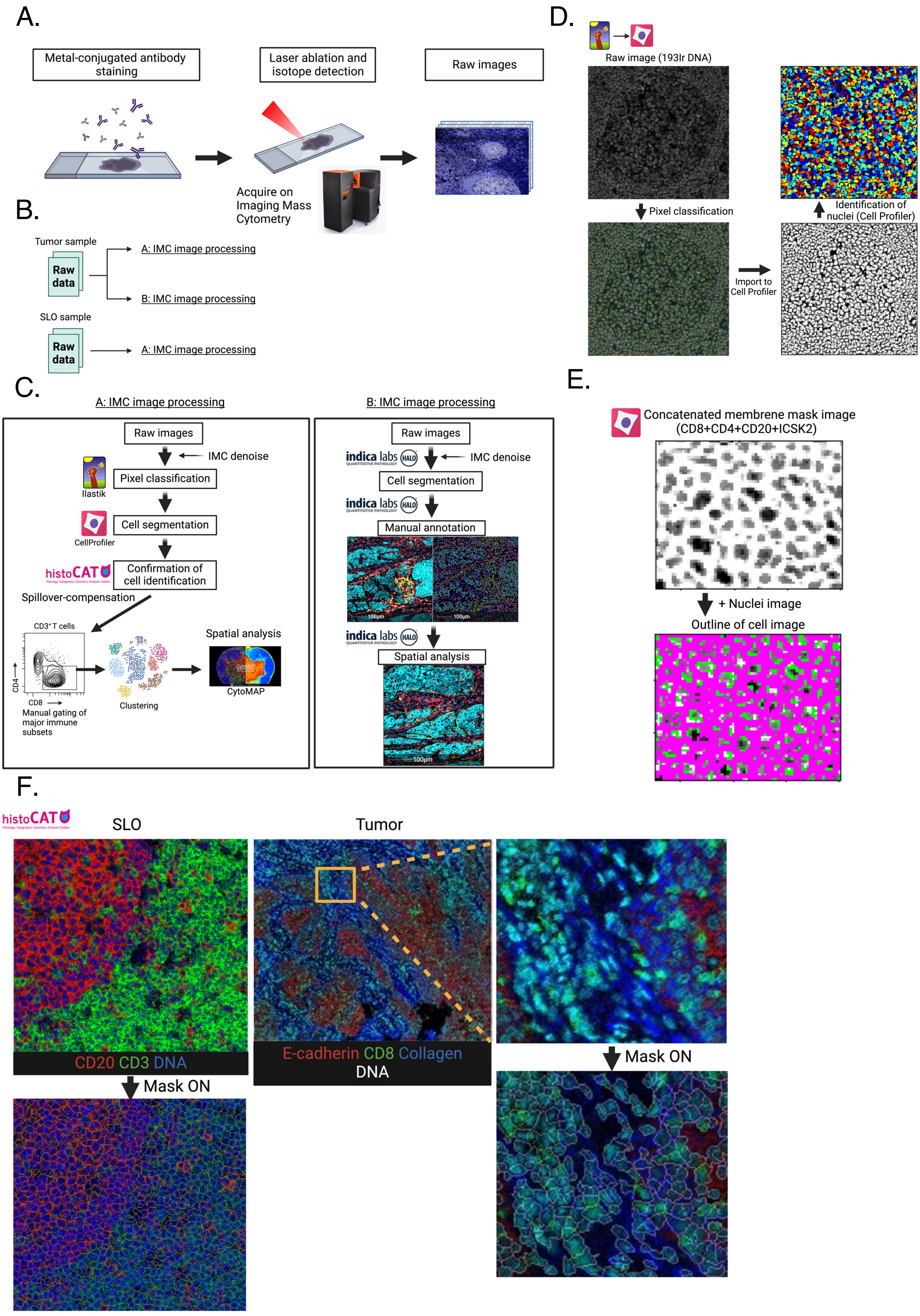

(A) Graphic summary of the data acquisition workflow used for IMC. (B) Schematic illustration of the cell identification workflow for tumors (A+B: IMC image processing) and SLOs (A: IMC image processing), respectively. (C) Each graphic workflow of A and B: IMC image processing. (D) Example of nuclei identification using A: IMC image processing. (E) Example of membrane mask for identified nuclei using A: IMC image processing. (F) Representative cell identification of each SLO (left) and tumor (right) after nuclei identification and membrane masking according to A: IMC image processing. The left images show the SLO IMC image, colored according to CD20 (red), CD3 (green), and DNA (blue). The right tumor IMC images are colored according to E-cadherin (red), CD8 (green), Collagen (blue), and DNA (white). (D, E, F) Staining patterns or single-cell masks were compared for all independent ROIs with similar results. (A, B, C, D, E, F) were created in BioRender. Kenro, T. (2024) BioRender.com/g70j848.

Supplementary Fig. 11. Comparison of the denoised IMC images versus immunofluorescence-stained images

A.

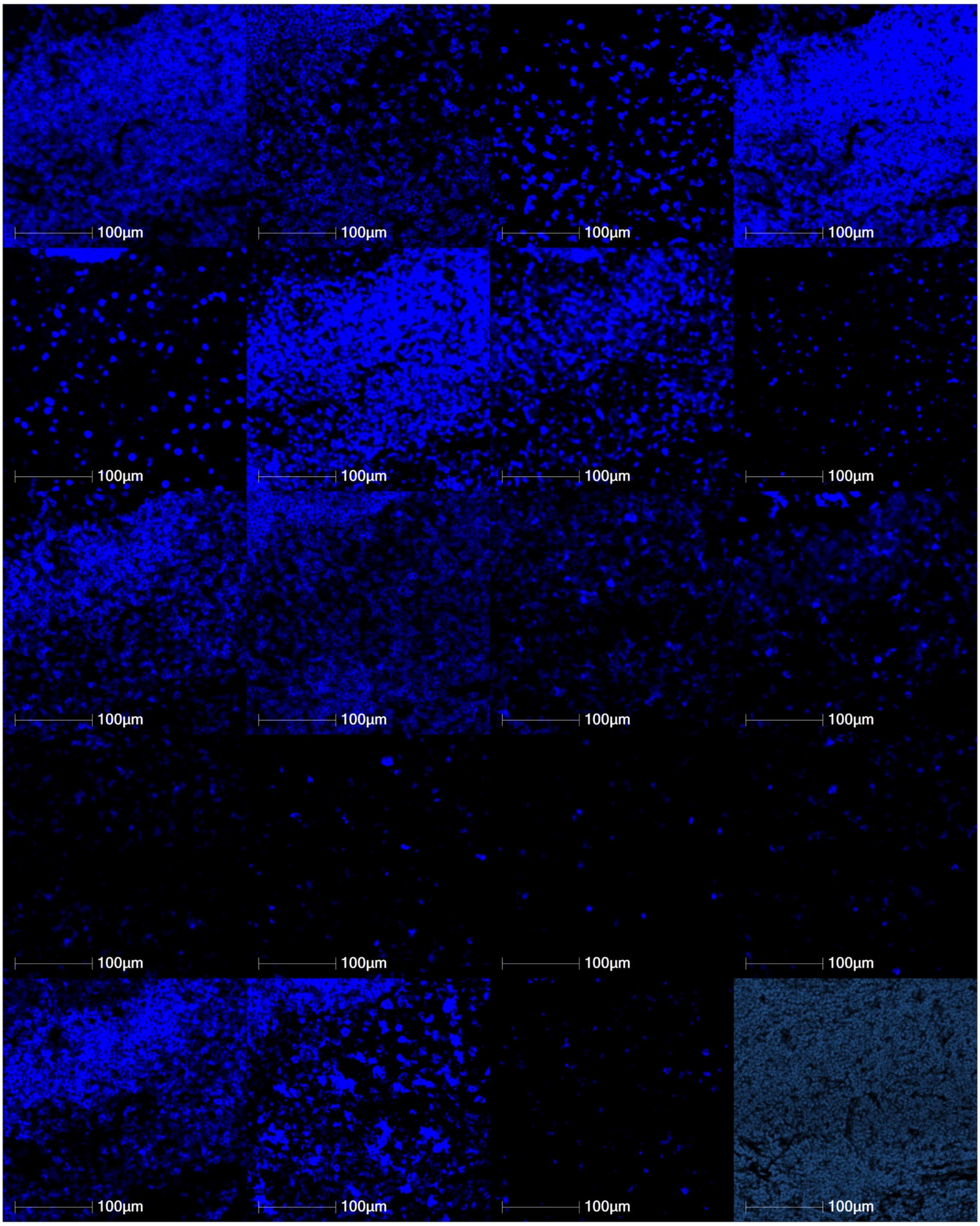

CD3	CD20	CD8	CD4
Ki67	TCF-1	TOX	FoxP3
CD45RO	CD45RA	CD39	PD-1
OX-40	TIM-3	LAG-3	CD25
CD69	HLADR	CD11c	DNA

B.

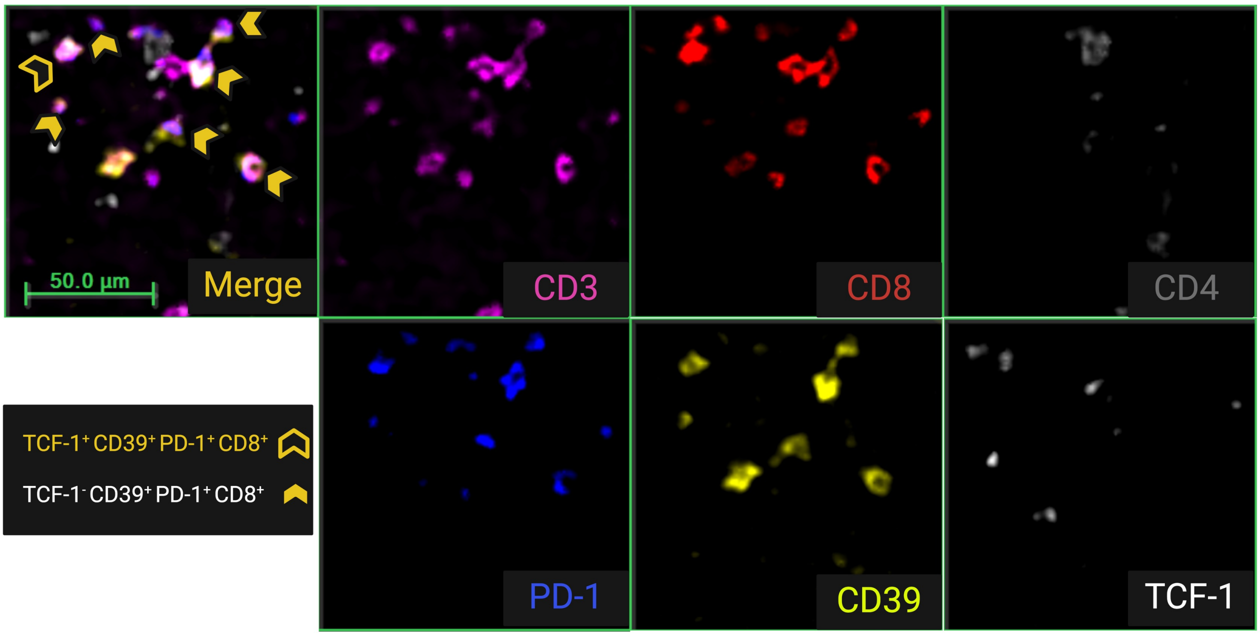

50.0 µm
Merge
CD3
CD8
CD4
TCF-1⁺ CD39⁺ PD-1⁺ CD8⁺
TCF-1⁻ CD39⁺ PD-1⁺ CD8⁺
PD-1
CD39
TCF-1

C.

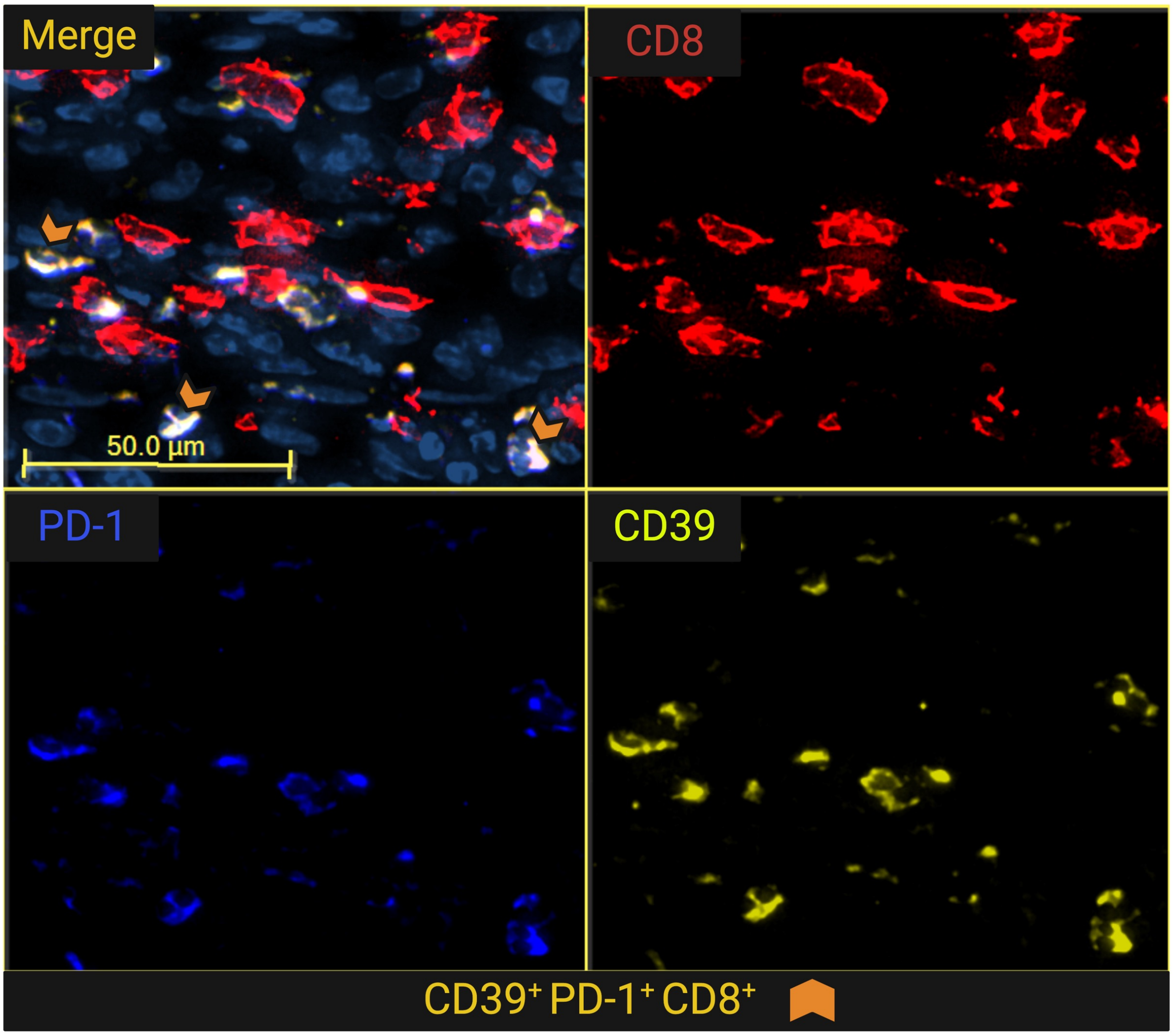

Merge
CD8
50.0 µm
PD-1
CD39
CD39⁺ PD-1⁺ CD8⁺

(A) The representative denoised images of each marker from a SLO-ROI. Each marker is color-coded in blue. (B) The identification of $CD39^{+}PD\text{-}1^{+}CD8^{+}$ T cells from a denoised tumor-ROI. Open arrowheads indicate $TCF\text{-}1^{+}CD39^{+}PD\text{-}1^{+}CD8^{+}$ T cells, while solid arrowheads indicate $TCF\text{-}1^{-}CD39^{+}PD\text{-}1^{+}CD8^{+}$ T cells. The denoised IMC image is pseudo-colored as follows: CD39 (green), PD-1 (blue), CD3 (red), CD4 (grey), TCF-1 (white) and CD3 (pink). (C) The representative immunofluorescence-stained images of CD8, PD-1, and CD39 from a tumor sample. The merged image also includes DAPI (nuclear) staining. Solid arrowheads indicate $CD39^{+}PD\text{-}1^{+}CD8^{+}$ T cells. The fluorescent image is pseudo-colored as follows: CD39 (green), PD-1 (blue), DAPI (dark blue) and CD8 (red). The sample (patient ID: KU37) for fluorescent immunostaining was selected from all 27 patients based on its classification as a hot tumor. (A, B) The quality of the denoised IMC images was compared for all independent ROIs with similar results. (B, C) were created in BioRender. Kenro, T. (2024) BioRender.com/e59f589.

Supplementary Fig. 12. Gating strategy for cell type identification using IMC

A.

Gating strategy for image processing A (SLO)

Immune subset	Gating phenotype
CD8+ T cells	CD3+, CD8+, CD4-, CD20- CD45+
Conventional CD4+ Tnaive cells	FoxP3-, CD45RA+, CD3+, CD8-, CD4+, CD20-, CD45+
Conventional CD4+ Tmemory cells	FoxP3-, CD45RA-, CD3+, CD8-, CD4+, CD20-, CD45+
PD-1+ Treg cells	FoxP3+, PD-1+, CD3+, CD8-, CD4+, CD20-, CD45+
PD-1- Treg cells	FoxP3+, PD-1-, CD3+, CD8-, CD4+, CD20-, CD45+
Antigen presenting cells	FoxP3+, PD-1-, CD3+, CD8-, CD4+, CD20-, CD45+
CD20+ B cells	CD20+, CD3-, CD45+

B.

Annotation strategy for image processing B

Immune subset	Annotated phenotype
TCF-1+CD39+PD-1+CD8+ T Cells	TCF-1+, CD39+, PD-1+, CD3+, CD8+, CD4-
TCF-1-CD39+PD-1+CD8+ T Cells	TCF-1-, CD39+, PD-1+, CD3+, CD8+, CD4-
CD8+ Tnaive cells	CD39-, PD-1-, CD45RA+, CD3+, CD8+, CD4-
CD8+ Tmemory cells	CD39-, PD-1-, CD45RA-, CD3+, CD8+, CD4-
Conventional CD4+ Tnaive cells	FoxP3-, CD45RA+, CD3+, CD8-, CD4+
Conventional CD4+ Tmemory cells	FoxP3-, CD45RA-, CD3+, CD8-, CD4+
PD-1+ Treg cells	FoxP3+, PD-1+, CD3+, CD8-, CD4+
PD-1- Treg cells	FoxP3+, PD-1-, CD3+, CD8-, CD4+
Monocytes or Macrophages	CD14+ or CD68+, CD3-, E -cadherin-, Pan-cytokerachin-
Tumor cell	E -cadherin+ or Pan-cytokerachin+, CD3-
CD20+ B cells	CD20+, CD3-

C.

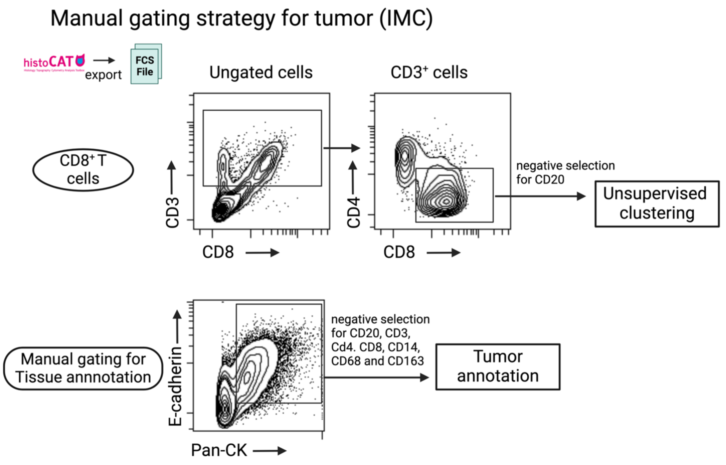

E.

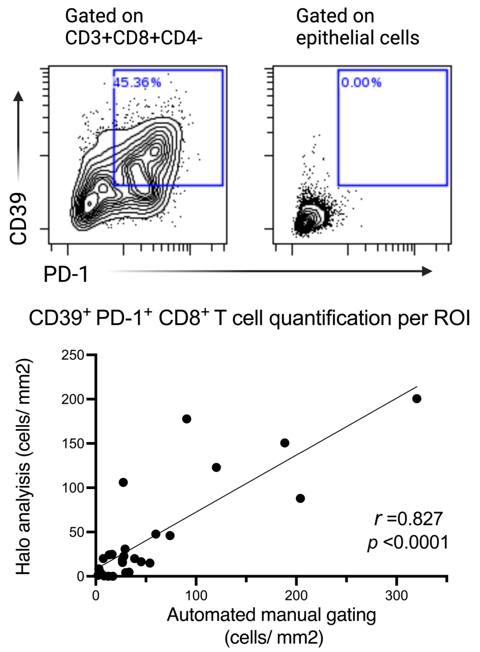

D.

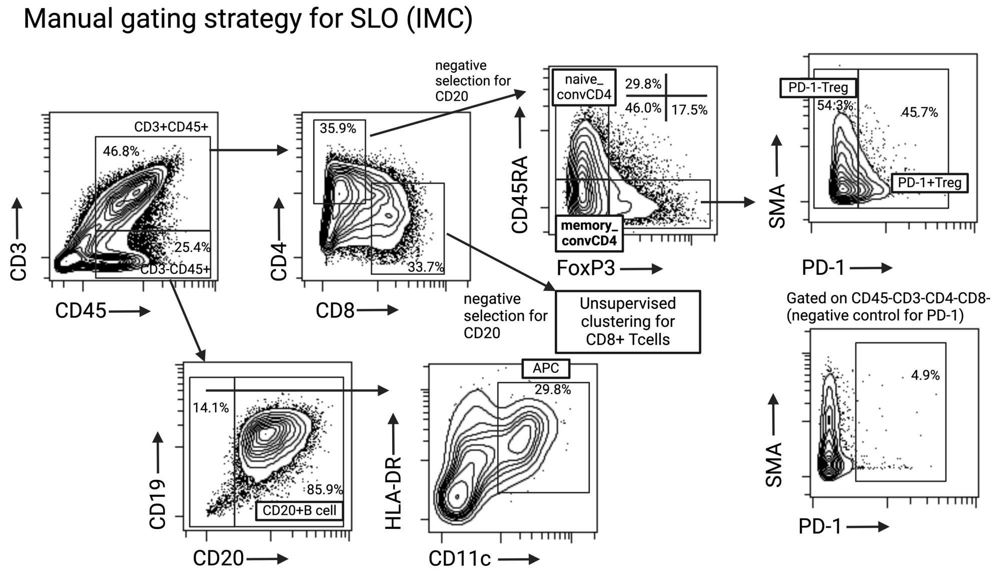

(A) Immune subsets identified using A: IMC image processing and gating panels to identify each subset. (B) Immune subsets identified using B: IMC image processing and gating panels to identify each subset. (C) Manual gating strategy of $CD8^+$ T cells ($CD3^+CD8^+CD4^-CD20^-$) within tumor-ROIs (top), and epithelial cells for tumor annotation (bottom). (D) Manual gating strategy for $CD8^+$ T cells, naïve conventional CD4 (convCD4), memory convCD4, PD-1^+ Treg, PD-1^- Treg and antigen presenting cells (APC) within SLO-ROIs. The gating plots in the bottom right show the negative control ($CD45^-CD3^-CD4^-CD20^-$) for PD-1. (E) The upper plots show an example of quantifying $CD39^+$PD-$1^+$$CD8^+$ T cells ($CD39^+$PD-$1^+$$CD3^+CD8^+CD4^-$) from normalized IMC data in Image processing A, based on negative control (tumor annotation). The lower plots compare the cell density of $CD39^+$PD-$1^+$$CD8^+$ T cells automatically gated with those quantified by HALO software; Pearson's correlations using a two-sided test (n=27, r=0.827, P<0.0001). (C, D) were created in BioRender. Kenro, T. (2024) BioRender.com/e59f589. Source data are provided as a Source Data file.

Supplementary Table 1. Patient characteristics.

**Characteristics**	**N=31**
Age	Median (range)
Years	72 (40-85)
Sex	N (%)
Male	27 (87)
Female	4 (13)
ECOG PS	
0	4 (13)
1	27 (87)
Unresectable	
Yes	31 (100)
No	0 (0)
Histology	
SCC	31 (100)
Brain metastases	
Yes	2 (6.5)
No	29 (93.5)
Previous therapy	
Surgery	10 (32.3)
Radiotherapy	19 (61.3)
Chemotherapy	30 (96.8)
Refractory or intolerant to platinum regimen	
Yes	30 (96.8)
No	1 (3.2)
Measurable lesions	
Yes	24 (77.4)
No	7 (22.6)
Available archival FFPE samples	N=27 (%)
Resection	8 (29.6)
Biopsy	19 (70.4)
PD-L1 CPS	N=27 (%)
CPS <1	2 (7.4)
1≦ CPS <5	2 (7.4)
5≦ CPS <10	4 (14.8)
10≦ CPS	19 (70.4)

Supplementary Table 2. List of tumor panel for imaging mass cytometry.

Metal	Antigen	Clone	Vendor	Catalog No	Dilution
141Pr	SMA	1A4	Fluidigm	3141017D	1:400
143Nd	Vimentin	D21H3	Fluidigm	3143027D	1:1600
142Nd	CD19	6OMP31	Fluidigm	3142014D	1:200
144Nd	CD14	EPR3653	Fluidigm	3144025D	1:1600
147Sm	CD163	EDHu-1	Fluidigm	3147021D	1:400
148Nd	Pan-Keratin	C11	Fluidigm	3148020D	1:200
150Nd	PD-L1	SP142	Fluidigm	3150033D	1:50
151Eu	OX40	Poly	Fluidigm	3151024D	1:100
152Sm	CD45	CD45-2B11	Fluidigm	3152016D	1:100
153Eu	LAG3	D2G4O	Fluidigm	3153028D	1:200
154Sm	TIM-3	D5D5R	Fluidigm	3154024D	1:800
155Gd	FoxP3	236A/E7	Fluidigm	3155016D	1:50
156Gd	CD4	EPR6855	Fluidigm	3156033D	1:200
158Gd	E-cadherin	24E10	Fluidigm	3158029D	1:400
159Tb	CD68	KP1	Fluidigm	3159035D	1:1600
161Dy	CD20	H1	Fluidigm	3161029D	1:800
162Dy	CD8a	C8/144B	Fluidigm	3162034D	1:800
163Dy	TCF-1	C63D9	CST	#85942	1:100
164Dy	Tox	E613Q	CST	#62886	1:400
165Ho	PD-1	D4W2J	CST	63815SF	1:100
166Er	CD45RA	HI100	Fluidigm	3166031D	1:1600
167Er	CD69	EPR21814	Abcam	ab234512	1:200
168Er	Ki-67	B56	Fluidigm	3168022D	1:200
169Tm	Collagen I	Poly	Fluidigm	3169023D	1:200
170Er	CD3	Poly	Fluidigm	3170019D	1:100
172Yb	CD39	EPR20627	Abcam	ab236038	1:50
173Yb	CD28	ERP22076	Abcam	ab243557	1:50
175Lu	CD25	EPR6452	Fluidigm	3175036D	1:50
195Pt	ICSK1		Fluidigm	201500	1:100
196Pt	ICSK2		Fluidigm	201500	1:100
198Pt	ICSK3		Fluidigm	201500	1:100

Supplementary Table 3. List of SLO panel for imaging mass cytometry.

Metal	Antigen	Clone	Vendor	Catalog No	Dilution
141Pr	SMA	1A4	Fluidigm	3141017D	1:400
143Nd	Vimentin	D21H3	Fluidigm	3143027D	1:1600
142Nd	CD19	6OMP31	Fluidigm	3142014D	1:200
144Nd	CD14	EPR3653	Fluidigm	3144025D	1:1600
147Sm	CD163	EDHu-1	Fluidigm	3147021D	1:400
148Nd	Pan-Keratin	C11	Fluidigm	3148020D	1:200
149Sm	CD11b	L291H4	Fluidigm	3149030D	1:100
150Nd	PD-L1	SP142	Fluidigm	3150033D	1:50
151Eu	OX40	Poly	Fluidigm	3151024D	1:100
152Sm	CD45	D9M8I	Fluidigm	3152018D	1:1600
153Eu	LAG3	D2G4O	Fluidigm	3153028D	1:200
154Sm	TIM-3	D5D5R	Fluidigm	3154024D	1:800
155Gd	FoxP3	236A/E7	Fluidigm	3155016D	1:50
156Gd	CD4	EPR6855	Fluidigm	3156033D	1:200
158Gd	E-cadherin	24E10	Fluidigm	3158029D	1:400
159Tb	CD68	KP1	Fluidigm	3159035D	1:1600
161Dy	CD20	H1	Fluidigm	3161029D	1:800
162Dy	CD8a	C8/144B	Fluidigm	3162034D	1:800
163Dy	TCF-1	C63D9	CST	#85942	1:100
164Dy	Tox	E613Q	CST	#62886	1:400
165Ho	PD-1	D4W2J	CST	63815SF	1:100
166Er	CD45RA	HI100	Fluidigm	3166031D	1:1600
167Er	CD69	EPR21814	Abcam	ab234512	1:200
168Er	Ki-67	B56	Fluidigm	3168022D	1:200
169Tm	Collagen I	Poly	Fluidigm	3169023D	1:200
170Er	CD3	Poly	Fluidigm	3170019D	1:100
171Yb	CD11c	D3V1E	CST	#93233	1:200
172Yb	CD39	EPR20627	Abcam	ab236038	1:50
173Yb	CD45RO	UCHL1	Fluidigm	3173016D	1:3200
174Yb	HLA-DR	LN3	Fluidigm	3174025D	1:1600
175Lu	CD25	EPR6452	Fluidigm	3175036D	1:50
195Pt	ICSK1		Fluidigm	201500	1:100
196Pt	ICSK2		Fluidigm	201500	1:100
198Pt	ICSK3		Fluidigm	201500	1:100

Supplementary Table 4. List of panel for mass flow cytometry.

Metal	Antigen	Clone	Vendor	Catalog No	Dilution
106Cd	CD45	HI30	Fluidigm	3106001B	1:100
110Cd	CD45	HI30	Fluidigm	3110001B	1:100
111Cd	CD45	HI30	Fluidigm	3111001B	1:100
112Cd	CD45	HI30	Fluidigm	3112001B	1:100
113Cd	CD45	HI30	Fluidigm	3113001B	1:100
114Cd	CD45	HI30	Fluidigm	3114001B	1:100
141Pr	CCR6	11A9	Fluidigm	3141014A	1:100
143Nd	CD45RA	HI100	Fluidigm	3143006B	1:100
145Nd	CD4	RPA-T4	Fluidigm	3145001B	1:100
146Nd	CD8	RPA-T8	Fluidigm	3146001B	1:100
147Sm	Ki67	Ki67	BioLegend	350523	1:100
149Sm	CD25	2A3	Fluidigm	3149010B	1:100
150Nd	OX-40	ACT35	Fluidigm	3150023B	1:100
152Sm	CD28	CD28.2	Biolegend	302937	1:100
153Eu	TIM-3	F38-2E2	Fluidigm	3153008B	1:100
154Sm	CD3	UCHT1	Fluidigm	3154003B	1:100
155Gd	TOX	REA473	Miltenyi Biotec	130-126-455	1:100
156Gd	CXCR3	G025H7	Fluidigm	3156004B	1:100
158Gd	CD27	L128	Fluidigm	3158010B	1:100
159Tb	FoxP3	259D/C7	Fluidigm	3159028A	1:100
160Gd	CD39	A1	Fluidigm	3160004B	1:100
161Dy	Tbet	4B10	Fluidigm	3161014B	1:100
162Dy	CD69	FN50	Fluidigm	3162001B	1:100
164Dy	CD45RO	UCHL1	Fluidigm	3164007B	1:100
165Ho	LAG-3	11C3C65	Fluidigm	3165037B	1:100
167Er	CCR7	G043H7	Fluidigm	3167009A	1:100
168Er	CD127	A019D5	Fluidigm	3168017B	1:100
169Tm	TCF-1	7F11A10	BioLegend	655202	1:100
170Er	CTLA-4	14D3	Fluidigm	3170005B	1:100
171Yb	CXCR5	RF8B2	Fluidigm	3171014B	1:100
172Yb	CX3CR1	2A9-1	Fluidigm	3172017B	1:100
173Yb	CXCR4	12G5	Fluidigm	3173001B	1:100
174Yb	Eomes	WD1928	eBioscience	14-4877-82	1:100
175Lu	anti_IgG4	HP6025	Southern Biotech	9200-01	1:50

Supplementary Table 5. List of key reagents

Reagent	Clone	Vendor	Catalog No	Dilution
For critical muliticolor FC assays				
Ki-67-BV421	Ki67	BioLegend	350506	1:50
Ki-67-PE/Cy7	Ki67	BioLegend	350526	1:50
CD4-PerCP/Cy5.5	SK3	BioLegend	344608	1:50
CD39-APC/Fire750	A1	BioLegend	328230	1:20
CD39-BV421	A1	BioLegend	328214	1:25
Eomes-FITC	WD1928	eBioscience	11-4877-42	1:20
Tox-PE	TXRX10	eBioscience	12-6502-82	1:20
Tbet-PE/Cy7	4B10	BioLegend	644824	1:50
CD14-BV510	M5E2	BioLegend	301842	1:50
CD8-BV605	SK1	BioLegend	344742	1:50
TIM-3-BV711	7D3	BD	565567	1:50
CD69-FITC	FN50	BioLegend	310904	1:20
TCF-1-PE	7F11A10	BioLegend	655208	1:20
OX-40-BV421	ACT35	BioLegend	350014	1:50
IFN-γ-BV711	4S.B3	BD	564039	1:50
TNF-FITC	Mab11	BD	554512	1:50
PD-1-PE	EH12.H7	Biolegend	329905	1:50
Mouse Anti-Human IgG4 Fc-AF647	HP6025	SouthernBiotech	9200-31	1:50
Mouse IgG1, κ Isotype Ctrl-BV421	-	BioLegend	400157	-
Mouse IgG1, κ Isotype Ctrl-PE	-	BioLegend	400111	-
Mouse IgG1, κ Isotype Ctrl-FITC	-	BioLegend	400107	-
Mouse IgG1, κ Isotype Ctrl-PE/Cy7	-	BioLegend	400125	-
Rat IgG2a, κ Isotype Ctrl-PE	-	BD	553930	-
Mouse IgG1, κ Isotype Ctrl-BV711	-	BioLegend	400167	-
Zombie Aqua™ Fixable Viability Kit	-	BioLegend	423101	-
Fixation/Permeabilization Diluent	-	eBioscience	00-5223-56	-
Fixation/Permeabilization Concentrate	-	eBioscience	00-5123-43	-
Permeabilization Buffer (10X)	-	eBioscience	00-8333-56	-
FcR Blocking Reagent, human	-	Miltenyi Biotec	130-059-901	-
For mass cytometry assays				
MaxPar X8 Antibody Labeling Kits	-	Fluidigm	201300	-
Cell-ID™ Intercalator-Ir	-	Fluidigm	201192B	-
Maxpar Cell Acquisition Solution	-	Fluidigm	201240	-
Maxpar Water	-	Fluidigm	201069	-
Maxpar PBS	-	Fluidigm	201058	-
Maxpar Cell Staining Buffer	-	Fluidigm	201068	-
Maxpar Nuclear Antigen Staining Buffer Set	-	Fluidigm	201063	-
EQ Four Element Calibration Beads	-	Fluidigm	201078	-
Cell-ID™ Intercalator-Rh	-	Fluidigm	201103A	-

Continued

Pierce™ 16% Formaldehyde (w/v), Methanol-free	-	Thermo Fisher	28906	-
Deoxyribonuclease 1, from Bovine Pancreas, Precrystalline	-	FUJIFILM	043-26773	-
Nivolumab	-	Sellek Chemicals	A2002	1:400
Ultra-LEAF™ Purified Human IgG4 Isotype Control Recombinant Antibody	QA16A15	Biolegend	403701	-
Candor PBS Antibody Stabilization solution	-	Candor Bioscience	131050	-
1M MgCl2	-	Nippon Gene	310-90361	-
30w/v% Albumin Solution, from Bovine Serum(BSA), Fatty Acid Free	-	Wako	017-22231	-
Antigen Unmasking Solution, Tris-Based	-	Vector Laboratories	H-3301-250	-
Critical IHC assays				
PD-L1 antibody (28-8) IHC antigen retrieval and detection panel	28-8	Abcam	ab236676	1:50
Anti-PD-1	NAT105	Abcam	ab52587	1:50
Anti-CD39	ERP20627	Abcam	ab223842	1:500
Anti-CD8	4B11	Leica Biosystems	PA0183	Prediluted
Goat anti-mouse IgG2b CF Dye 647	Polyclonal	Biotium	20272-1	1:800
Goat anti-mouse IgG1 CF Dye 488	Polyclonal	Biotium	20246-1	1:800
Goat anti-rabbit IgG CF Dye 568	Polyclonal	Biotium	20103-1	1:800
EnVision+ Dual Link System-HRP (DAB+)	-	Agilent Technologies	K406311	Prediluted
Anti-CD3	F7.2.38	Agilent Technologies	M7254	1:20
Anti-CD20cy	L26	Agilent Technologies	IS604	Prediluted
Anti-PNA	MECA79R	Novus	NBP2-78792	1:100
Protein Block Serum-Free	-	Agilent Technologies	X0909	-
Antigen Retrieval Buffer (100X EDTA Buffer, pH 8.0)	-	Abcam	ab93680	-
