# Supplementary material for: Spatial dynamics of CD39+CD8+ exhausted T cell reveal tertiary lymphoid structures-mediated response to PD-1 blockade in esophageal cancer
Source: Nat Commun. 2024 Oct 19;15:9033. doi: 10.1038/s41467-024-53262-w (PMC11490492; doi:10.1038/s41467-024-53262-w)
Supplement: Supplementary file 3 — Reporting Summary [file 41467_2024_53262_MOESM3_ESM.pdf]

nature portfolio

Corresponding author(s): Eishi Baba
NCOMMS-23-61103B

Last updated by author(s): Aug 28, 2024

Reporting Summary

Nature Portfolio wishes to improve the reproducibility of the work that we publish. This form provides structure for consistency and transparency in reporting. For further information on Nature Portfolio policies, see our Editorial Policies and the Editorial Policy Checklist.

Statistics

For all statistical analyses, confirm that the following items are present in the figure legend, table legend, main text, or Methods section.

n/a	Confirmed	
☐	☒	The exact sample size (*n*) for each experimental group/condition, given as a discrete number and unit of measurement
☐	☒	A statement on whether measurements were taken from distinct samples or whether the same sample was measured repeatedly
☐	☒	The statistical test(s) used AND whether they are one- or two-sided<br>*Only common tests should be described solely by name; describe more complex techniques in the Methods section.*
☐	☒	A description of all covariates tested
☐	☒	A description of any assumptions or corrections, such as tests of normality and adjustment for multiple comparisons
☐	☒	A full description of the statistical parameters including central tendency (e.g. means) or other basic estimates (e.g. regression coefficient) AND variation (e.g. standard deviation) or associated estimates of uncertainty (e.g. confidence intervals)
☐	☒	For null hypothesis testing, the test statistic (e.g. *F*, *t*, *r*) with confidence intervals, effect sizes, degrees of freedom and *P* value noted<br>*Give P values as exact values whenever suitable.*
☒	☐	For Bayesian analysis, information on the choice of priors and Markov chain Monte Carlo settings
☐	☒	For hierarchical and complex designs, identification of the appropriate level for tests and full reporting of outcomes
☐	☒	Estimates of effect sizes (e.g. Cohen's *d*, Pearson's *r*), indicating how they were calculated

Our web collection on statistics for biologists contains articles on many of the points above.

Software and code

Policy information about availability of computer code

Data collection	CyTOF software (version 7.0.8493, Standard Biotools) for IMC data. Attune NxT Flow Cytometer (Thermo Fisher Scientific) and CyTOF software (version 7.0.8493, Standard Biotools) for human peripheral mononuclear cell data. BZ-X710 (KEYENCE, software BZ-H3A) for HE and immunohistochemical staining. Leica DMi8 THUNDER imaging system (Leica, Weztlar, Germany) for immmunofluorescence staining.
Data analysis	For validation of IMC staining and IMC data processing, MCD Viewer (v1.0.560.6, Standard Biotools) was used. For segmentation and single cell feature extraction, Cellprofiler version 4.2.1, ilastik 1.3.3post1 and histoCAT version 4.2.1 were used. For IMC denoise, deep learning models were implemented with standard libraries of Python (3.6.13), TensorFlow (2.2.0, Google), and Keras (2.3.1, Google). The IMC-denoise package is available through https://github.com/PENGLU-WashU/IMC_Denoise. For multicolor flowcytometry analysis and IMC data processing, the FlowJo software (version 10.7.1) was used. For both IMC and MC data analysis, Cytobank (https://cytobank.org) was used. For IMC data analysis, HALO image analysis software (v3.5.3577.265, Indica Labs) and CytoMAP (version 1.4.21) were used. Statistical analyses and figure generation were performed using the Graph pad Prism v9.5.1 and JMP pro 16 software.

For manuscripts utilizing custom algorithms or software that are central to the research but not yet described in published literature, software must be made available to editors and reviewers. We strongly encourage code deposition in a community repository (e.g. GitHub). See the Nature Portfolio guidelines for submitting code & software for further information.

Data

Policy information about availability of data

All manuscripts must include a data availability statement. This statement should provide the following information, where applicable:

- Accession codes, unique identifiers, or web links for publicly available datasets
- A description of any restrictions on data availability
- For clinical datasets or third party data, please ensure that the statement adheres to our policy

The MC and IMC data generated in this study are publicly available in the Zenodo database under the accession code https://doi.org/10.5281/zenodo.11421121 for academic non-commercial research. The MC and IMC data generated in this study are also provided in the Supplementary Information/Source Data file. The HNSCC MC dataset can be downloaded from https://doi.org/10.17632/2zgppyr2rr.1. All remaining data are included in the Supplementary Information or available from the authors, as are unique reagents used in this Article. The raw numbers for charts and graphs are available in the Source Data file whenever possible. Source data are provided with this paper.

Research involving human participants, their data, or biological material

Policy information about studies with human participants or human data. See also policy information about sex, gender (identity/presentation), and sexual orientation and race, ethnicity and racism.

Reporting on sex and gender	Information on sex was collected from medical records. Information on gender was not collected. Sex or gender were not considered in the study design as it was not relevant to our research objectives.
Reporting on race, ethnicity, or other socially relevant groupings	Analyses based on race, ethnicity, or other socially relevant groupings were not performed.
Population characteristics	Please refer to the Methods section and Table S1.
Recruitment	From March 2020 to July 2021, a total of 31 patients with unresectable or advanced ESCC (median age 72 years, range 40-85) were prospectively enrolled for nivolumab treatment (240 mg/patient every 2 weeks until disease progression, death, or unacceptable toxicity). Inclusion criteria were as follows: histologically confirmed ESCC, a baseline Eastern Cooperative Oncology Group performance status of 0–1, resistance or intolerance to fluoropyrimidine-based and platinum-based chemotherapy, age over 20 years, and no prior immunotherapy.<br><br>Patients were required to have a good performance status (0-1), which may result in a study population with a better prognosis than typical clinical presentations. On the other hand, patients without measurable lesions were also eligible, and the assessment of treatment effect was conducted differently from standard clinical trials. These selection biases could influence the effect of prognostic biomarkers. Additionally, the tumor samples used were archival, which may not fully reflect the microenvironment just before ICB treatment. Furthermore, this study was a prospective observational study, not an interventional one, and five cases in which the effect of Nivolumab could not be strictly evaluated due to these limitations were included. This limitation could affect the results.
Ethics oversight	Ethical approval was centrally granted by the Ethics Committee of Kyushu University Hospital (Approval No. 2023-17) and by the respective Ethics Committees of the following institutions: Japan Community Healthcare Organization Kyushu Hospital, NHO Kyushu Medical Center, Hamanomachi Hospital, Fukuoka Wajiro Hospital, and National Kyushu Cancer Center.

Note that full information on the approval of the study protocol must also be provided in the manuscript.

Field-specific reporting

Please select the one below that is the best fit for your research. If you are not sure, read the appropriate sections before making your selection.

[x] Life sciences [ ] Behavioural & social sciences [ ] Ecological, evolutionary & environmental sciences

For a reference copy of the document with all sections, see nature.com/documents/nr-reporting-summary-flat.pdf

Life sciences study design

All studies must disclose on these points even when the disclosure is negative.

Sample size	The initial target number of samples was set at 30 cases, meeting the eligibility criteria as described in the Methods section. Ultimately, 31 cases were prospectively enrolled. The target of 30 cases was based on previous literature (Alexander C. Huang et al., Nature, 2017; n=29), suggesting this sample size would be sufficient for analysis. No formal statistical analysis was performed to determine the sample size.
Data exclusions	In this study, to minimize the impact of covariates, eligibility criteria were predefined. Of the 31 enrolled cases, one case was excluded from the analysis, except for the peripheral blood cytokine analysis, because it was neither refractory to nor intolerant of fluoropyrimidine and platinum-based chemotherapy.<br>In the IMC analysis, 27 out of 30 eligible cases were analyzed. Pre-treatment FFPE samples for the remaining 3 patients were unavailable because two were held by other hospitals, and one was non-tumorous. In the peripheral blood analysis, two cases were missing samples at the two-week post-ICB administration point due to patient refusal and COVID-19 restrictions. Peripheral blood samples post-ICB

administration were collected optionally in cases of disease progression (PD), and 19 such cases were successfully collected. Additionally, 13 independent cases with TLS were identified, but one case was excluded from the analysis of TLS composition because the TLS was too small for proper analysis.
All available samples were successfully utilized in our study.

Replication	All figures presented with error bars were derived from multiple independent patient-derived samples, or multiple independent cell types in a patient sample (Fig.S7F) . The exact numbers are provided in the figures or figure legends.<br>Specifically, in the IMC analysis, all cell types, clusters, their spatial interactions, and associations with treatment outcomes were identified using tumor tissues from 27 independent patients or lymph nodes from 3 independent patients, out of a total of 31 enrolled cases. Analyses related to TLS were conducted on 12 or 13 independent cases. Although the IMC analysis of lymph nodes was limited to 3 independent cases, the presence of CD39+ Tpex cells in the lymph nodes was reliably reproduced in a public mass flow cytometry dataset. Similarly, the strong correlation between the abundance of CD39+ Tpex cells and CD39+PD-1+CD8+ T cells within the tumor was also reproduced in the same dataset.<br>The primary analysis of peripheral blood was conducted on 30 independent cases, and cytokine analysis was performed on 18 independent cases, including one ineligible case. The MC analysis was conducted on 6 independent cases, and similar results were confirmed through FC analysis.
Randomization	Randomization was not necessary for case registration due to the objectives of this study and was therefore not performed. For the selection of ROIs in TLS and tumor areas, random selection was conducted based on predefined criteria. Additionally, while 6 independent samples for MC analysis were randomly selected, these were not intended for use in survival analysis. Similarly, 18 individuals were randomly selected for cytokine analysis.
Blinding	The selection of ROIs in IMC, as well as data acquisition and analysis in both IMC and FC/MC, were performed without blinding the operators to the treatment outcomes. The visualization and threshold settings of IMC markers were ultimately reviewed by a pathologist who was blinded to the clinical data.

Reporting for specific materials, systems and methods

We require information from authors about some types of materials, experimental systems and methods used in many studies. Here, indicate whether each material, system or method listed is relevant to your study. If you are not sure if a list item applies to your research, read the appropriate section before selecting a response.

Materials & experimental systems

n/a	Involved in the study
☐	☒ Antibodies
☒	☐ Eukaryotic cell lines
☒	☐ Palaeontology and archaeology
☒	☐ Animals and other organisms
☒	☐ Clinical data
☒	☐ Dual use research of concern
☒	☐ Plants

Methods

n/a	Involved in the study
☒	☐ ChIP-seq
☐	☒ Flow cytometry
☒	☐ MRI-based neuroimaging

Antibodies

Antibodies used	Please refer to Supplementary Tables 2, 3, 4 and 5 for the list of antibodies used in analyses.<br><br>The following anti-human primary antibodies for multicolor flow cytometry were used: Ki-67-BV421 and Ki-67-PE/Cy7 (BioLegend, Clone Ki67, Cat No. 350506, 350526, 1:50), CD4-PerCP/Cy5.5 (BioLegend, Clone SK3, Cat No. 344608, 1:50), CD39-APC/Fire750 and CD39-BV421 (BioLegend, Clone A1, Cat No. 328230, 328214, 1:20, 1:25), Eomes-FITC (eBioscience, Clone WD1928, Cat No. 11-4877-42, 1:20), Tox-PE (eBioscience, Clone TXRX10, Cat No. 12-6502-82, 1:20), Tbet-PE/Cy7 (BioLegend, Clone 4B10, Cat No. 644824, 1:50), CD14-BV510 (BioLegend, Clone M5E2, Cat No. 301842, 1:50), CD8-BV605 (BioLegend, Clone SK1, Cat No. 344742, 1:50), TIM-3-BV711 (BD, Clone 7D3, Cat No. 565567, 1:50), CD69-FITC (BioLegend, Clone FN50, Cat No. 310904, 1:20), TCF-1-PE (BioLegend, Clone 7F11A10, Cat No. 655208, 1:20), OX-40-BV421 (BioLegend, Clone ACT35, Cat No. 350014, 1:50), IFN-γ-BV711 (BD, Clone 4S.B3, Cat No. 564039, 1:50), TNF-FITC (BD, Clone Mab11, Cat No. 554512, 1:50), Mouse IgG1, κ Isotype Ctrl-BV421 (BioLegend, Cat No. 400157), Mouse IgG1, κ Isotype Ctrl-PE (BioLegend, Cat No. 400111), Mouse IgG1, κ Isotype Ctrl-FITC (BioLegend, Cat No. 400107), Mouse IgG1, κ Isotype Ctrl-PE/Cy7 (BioLegend, Cat No. 400125), Rat IgG2a, κ Isotype Ctrl-PE (BD, Cat No. 553930), and Mouse IgG1, κ Isotype Ctrl-BV711 (BioLegend, Cat No. 400167).<br><br>The following primary antibodies were used for IMC: SMA-141Pr (Fluidigm, Clone 1A4, Cat No. 3141017D, 1:400), Vimentin-143Nd (Fluidigm, Clone D21H3, Cat No. 3143027D, 1:1600), CD19-142Nd (Fluidigm, Clone 6OMP31, Cat No. 3142014D, 1:200), CD14-144Nd (Fluidigm, Clone EPR3653, Cat No. 3144025D, 1:1600), CD163-147Sm (Fluidigm, Clone EDHu-1, Cat No. 3147021D, 1:400), Pan-Keratin-148Nd (Fluidigm, Clone C11, Cat No. 3148020D, 1:200), CD11b-149Sm (Fluidigm, Clone L291H4, Cat No. 3149030D, 1:100), PD-L1-150Nd (Fluidigm, Clone SP142, Cat No. 3150033D, 1:50), OX40-151Eu (Fluidigm, Polyclonal, Cat No. 3151024D, 1:100), CD45-152Sm (Fluidigm, Clone D9M8I, Cat No. 3152018D, 1:1600), CD45-152Sm (Fluidigm, Clone CD45-2B11, Cat No. 3152016D, 1:100), LAG3-153Eu (Fluidigm, Clone D2G4O, Cat No. 3153028D, 1:200), TIM-3-154Sm (Fluidigm, Clone D5D5R, Cat No. 3154024D, 1:800), FoxP3-155Gd (Fluidigm, Clone 236A/E7, Cat No. 3155016D, 1:50), CD4-156Gd (Fluidigm, Clone EPR6855, Cat No. 3156033D, 1:200), E-cadherin-158Gd (Fluidigm, Clone 24E10, Cat No. 3158029D, 1:400), CD68-159Tb (Fluidigm, Clone KP1, Cat No. 3159035D,

1:1600), CD20-161Dy (Fluidigm, Clone H1, Cat No. 3161029D, 1:800), CD8a-162Dy (Fluidigm, Clone C8/144B, Cat No. 3162034D, 1:800), TCF-1-163Dy (CST, Clone C63D9, Cat No. #85942, 1:100), Tox-164Dy (CST, Clone E613Q, Cat No. #62886, 1:400), PD-1-165Ho (CST, Clone D4W2J, Cat No. 63815SF, 1:100), CD45RA-166Er (Fluidigm, Clone HI100, Cat No. 3166031D, 1:1600), CD69-167Er (Abcam, Clone EPR21814, Cat No. ab234512, 1:200), Ki-67-168Er (Fluidigm, Clone B56, Cat No. 3168022D, 1:200), Collagen I-169Tm (Fluidigm, Polyclonal, Cat No. 3169023D, 1:200), CD3-170Er (Fluidigm, Polyclonal, Cat No. 3170019D, 1:100), CD11c-171Yb (CST, Clone D3V1E, Cat No. #93233, 1:200), CD39-172Yb (Abcam, Clone EPR20627, Cat No. ab236038, 1:50), CD45RO-173Yb (Fluidigm, Clone UCHL1, Cat No. 3173016D, 1:3200), CD28-173Yb (Abcam, Clone ERP22076, Cat No. ab243557, 1:50), HLA-DR-174Yb (Fluidigm, Clone LN3, Cat No. 3174025D, 1:1600), CD25-175Lu (Fluidigm, Clone EPR6452, Cat No. 3175036D, 1:50), ICSK1-195Pt (Fluidigm, Cat No. 201500, 1:100), ICSK2-196Pt (Fluidigm, Cat No. 201500, 1:100), ICSK3-198Pt (Fluidigm, Cat No. 201500, 1:100).

The following primary antibodies and detection systems were used for critical IHC assays: PD-L1 antibody (28-8) IHC antigen retrieval and detection panel (Abcam, Clone 28-8, Cat No. ab236676, 1:50), Anti-PD-1 (Abcam, Clone NAT105, Cat No. ab52587, 1:50), Anti-CD39 (Abcam, Clone ERP20627, Cat No. ab223842, 1:500), Anti-CD8 (Leica Biosystems, Clone 4B11, Cat No. PA0183, Prediluted), Goat anti-mouse IgG2b CF Dye 647 (Biotium, Polyclonal, Cat No. 20272-1, 1:800), Goat anti-mouse IgG1 CF Dye 488 (Biotium, Polyclonal, Cat No. 20246-1, 1:800), Goat anti-rabbit IgG CF Dye 568 (Biotium, Polyclonal, Cat No. 20103-1, 1:800), EnVision+ Dual Link System-HRP (DAB+) (Agilent Technologies, Cat No. K406311, Prediluted), Anti-CD3 (Agilent Technologies, Clone F7.2.38, Cat No. M7254, 1:20), Anti-CD20cy (Agilent Technologies, Clone L26, Cat No. IS604, Prediluted), and Anti-PNA (Novus, Clone MECA79R, Cat No. NBP2-78792, 1:100).

The following primary antibodies were used for MC: CD45-106Cd (Fluidigm, Clone HI30, Cat No. 3106001B, 1:100), CD45-110Cd (Fluidigm, Clone HI30, Cat No. 3110001B, 1:100), CD45-111Cd (Fluidigm, Clone HI30, Cat No. 3111001B, 1:100), CD45-112Cd (Fluidigm, Clone HI30, Cat No. 3112001B, 1:100), CD45-113Cd (Fluidigm, Clone HI30, Cat No. 3113001B, 1:100), CD45-114Cd (Fluidigm, Clone HI30, Cat No. 3114001B, 1:100), CCR6-141Pr (Fluidigm, Clone 11A9, Cat No. 3141014A, 1:100), CD45RA-143Nd (Fluidigm, Clone HI100, Cat No. 3143006B, 1:100), CD4-145Nd (Fluidigm, Clone RPA-T4, Cat No. 3145001B, 1:100), CD8-146Nd (Fluidigm, Clone RPA-T8, Cat No. 3146001B, 1:100), Ki67-147Sm (BioLegend, Clone Ki67, Cat No. 350523, 1:100), CD25-149Sm (Fluidigm, Clone 2A3, Cat No. 3149010B, 1:100), OX-40-150Nd (Fluidigm, Clone ACT35, Cat No. 3150023B, 1:100), CD28-152Sm (BioLegend, Clone CD28.2, Cat No. 302937, 1:100), TIM-3-153Eu (Fluidigm, Clone F38-2E2, Cat No. 3153008B, 1:100), CD3-154Sm (Fluidigm, Clone UCHT1, Cat No. 3154003B, 1:100), TOX-155Gd (Miltenyi Biotec, Clone REA473, Cat No. 130-126-455, 1:100), CXCR3-156Gd (Fluidigm, Clone G025H7, Cat No. 3156004B, 1:100), CD27-158Gd (Fluidigm, Clone L128, Cat No. 3158010B, 1:100), FoxP3-159Tb (Fluidigm, Clone 259D/C7, Cat No. 3159028A, 1:100), CD39-160Gd (Fluidigm, Clone A1, Cat No. 3160004B, 1:100), Tbet-161Dy (Fluidigm, Clone 4B10, Cat No. 3161014B, 1:100), CD69-162Dy (Fluidigm, Clone FN50, Cat No. 3162001B, 1:100), CD45RO-164Dy (Fluidigm, Clone UCHL1, Cat No. 3164007B, 1:100), LAG-3-165Ho (Fluidigm, Clone 11C3C65, Cat No. 3165037B, 1:100), CCR7-167Er (Fluidigm, Clone G043H7, Cat No. 3167009A, 1:100), CD127-168Er (Fluidigm, Clone A019D5, Cat No. 3168017B, 1:100), TCF-1-169Tm (BioLegend, Clone 7F11A10, Cat No. 655202, 1:100), CTLA-4-170Er (Fluidigm, Clone 14D3, Cat No. 3170005B, 1:100), CXCR5-171Yb (Fluidigm, Clone RF8B2, Cat No. 3171014B, 1:100), CX3CR1-172Yb (Fluidigm, Clone 2A9-1, Cat No. 3172017B, 1:100), CXCR4-173Yb (Fluidigm, Clone 12G5, Cat No. 3173001B, 1:100), Eomes-174Yb (eBioscience, Clone WD1928, Cat No. 14-4877-82, 1:100), anti-IgG4-175Lu (Southern Biotech, Clone HP6025, Cat No. 9200-01, 1:50).

Validation

For information on antibody validation related to MC and IMC, please refer to the Methods section of the manuscript. The quality validation of commercially available antibodies used for MC and IMC is generally provided and ensured by the manufacturer (Standard Biotools). In IMC, both commercially available and newly labeled antibodies were tested prior to use on tonsil or tumor tissue via IMC analysis to ensure accurate performance under the appropriate conditions and concentrations. Specifically, for newly labeled antibodies, staining performance was confirmed via IHC prior to labeling. CD45-152Sm (CD45-2B11) and CD28-173Yb (EPR22076) used in IMC were excluded from the analysis due to weak staining performance.

For DAB immunohistochemistry, fluorescent immunohistochemistry, and flow cytometry, only commercially available antibodies from manufacturers such as Abcam, Novus, BioLegend, BD, eBioscience, Agilent Technologies, Biotium, and Leica Biosystems were used, with quality validation provided and ensured by the manufacturer. The validation of PD-1 using Nivolumab in peripheral blood and MC is described in the Methods section. For fluorescent immunohistochemistry, antibodies pre-validated for concentration by a pathologist were used. Flow cytometry performance was confirmed under appropriate conditions and concentrations using human PBMCs prior to use, and for DAB immunohistochemistry, validation was conducted using tonsil and tumor tissues.

Plants

Seed stocks

Report on the source of all seed stocks or other plant material used. If applicable, state the seed stock centre and catalogue number. If plant specimens were collected from the field, describe the collection location, date and sampling procedures.

Novel plant genotypes

Describe the methods by which all novel plant genotypes were produced. This includes those generated by transgenic approaches, gene editing, chemical/radiation-based mutagenesis and hybridization. For transgenic lines, describe the transformation method, the number of independent lines analyzed and the generation upon which experiments were performed. For gene-edited lines, describe the editor used, the endogenous sequence targeted for editing, the targeting guide RNA sequence (if applicable) and how the editor was applied.

Authentication

Describe any authentication procedures for each seed stock used or novel genotype generated. Describe any experiments used to assess the effect of a mutation and, where applicable, how potential secondary effects (e.g. second site T-DNA insertions, mosiacism, off-target gene editing) were examined.

Flow Cytometry

Plots

Confirm that:

☒ The axis labels state the marker and fluorochrome used (e.g. CD4-FITC).

☒ The axis scales are clearly visible. Include numbers along axes only for bottom left plot of group (a 'group' is an analysis of identical markers).

☒ All plots are contour plots with outliers or pseudocolor plots.

☒ A numerical value for number of cells or percentage (with statistics) is provided.

Methodology

Sample preparation	PBMCs from patients and healthy donors were isolated via Ficoll gradient centrifugation and stored at −80°C. PBMCs were thawed and stained using a Zombie Aqua Fixable Viability kit (BioLegend). Prior to PD-1 staining, Fc receptor blocking reagent was used and incubated for 15 minutes. PD-1 was then detected using anti-human IgG4-Alexa Fluor 647 (HP6025, Southern Biotec) after saturation with nivolumab. Cells were then incubated for 30 minutes with fluorochrome-conjugated antibodies against surface proteins of interest. For intracellular staining, the cells were fixed for 30 minutes, permeabilized using Fixation/Permeabilization concentrate and Fixation/Permeabilization diluent (eBioscience), and incubated for 30 minutes with fluorochrome-conjugated antibodies against intracellular proteins.
Instrument	Attune NxT Flow Cytometer (Thermo Fisher Scientific)
Software	FlowJo software (version 10.7.1)
Cell population abundance	For the detection of proliferative CD39+ Tpex cells, approximately 2 million frozen PBMCs per sample were used for all samples, yielding data from a maximum of 250,000 cells/sample to a minimum of 40,990 cells/sample.
Gating strategy	Lymphocyte gating was determined based on their position in the SSC-A/FSC-A plot, and doublets were excluded using FSC-W/FSC-H and SSC-W/SSC-H plots. Subsequently, BV510+ cells (dead cells and CD14+ cells) and CD4+ cells were negatively selected to gate CD8+ T cells. PD-1 gating was performed using a negative control of cells not saturated with Nivolumab, while thresholds for other markers were set using the following isotype controls: Mouse IgG1, κ Isotype Ctrl-BV421 (BioLegend, Cat No. 400157), Mouse IgG1, κ Isotype Ctrl-PE (BioLegend, Cat No. 400111), Mouse IgG1, κ Isotype Ctrl-FITC (BioLegend, Cat No. 400107), Mouse IgG1, κ Isotype Ctrl-PE/Cy7 (BioLegend, Cat No. 400125), Rat IgG2a, κ Isotype Control-PE (BD, Cat No. 553930), and Mouse IgG1, κ Isotype Ctrl-BV711 (BioLegend, Cat No. 400167).

☒ Tick this box to confirm that a figure exemplifying the gating strategy is provided in the Supplementary Information.
